# Mental health of clinical staff working in high-risk epidemic and pandemic health emergencies a rapid review of the evidence and living meta-analysis

**DOI:** 10.1007/s00127-020-01990-x

**Published:** 2020-11-27

**Authors:** Vaughan Bell, Dorothy Wade

**Affiliations:** 1 Research Department of Clinical, Educational and Health Psychology, University College London, London, UK; 2 South London and Maudsley NHS Foundation Trust, London, UK; 3 University College London Hospitals Critical Care Department, London, UK; 4 Research Department of Behavioural Science and Health, University College London, London, UK

**Keywords:** COVID-19, Mental health, Staff, Depression, Anxiety, PTSD

## Abstract

**Purpose:**

The SARS-CoV-2 / COVID-19 pandemic has raised concerns about the potential mental health impact on frontline clinical staff. However, given that poor mental health is common in acute medical staff, we aimed to estimate the additional burden of work involving high exposure to infected patients.

**Methods:**

We report a rapid review, meta-analysis, and living meta-analysis of studies using validated measures from outbreaks of COVID-19, Ebola, H1N1 influenza, Middle East respiratory syndrome (MERS), and severe acute respiratory syndrome (SARS).

**Results:**

A random effects meta-analysis found that high-exposure work is not associated with an increased prevalence of above cut-off scoring (anxiety: RR = 1.30, 95% CI 0.87–1.93, Total *N* = 12,473; PTSD symptoms: RR = 1.16, 95% CI 0.75–1.78, Total *N* = 6604; depression: RR = 1.50, 95% CI 0.57–3.95, Total *N* = 12,224). For continuous scoring, high-exposure work was associated with only a small additional burden of acute mental health problems compared to low-exposure work (anxiety: SMD = 0.16, 95% CI 0.02–0.31, Total *N* = 6493; PTSD symptoms: SMD = 0.20, 95% CI 0.01–0.40, Total *N* = 5122; depression: SMD = 0.13, 95% CI -0.04–0.31, Total *N* = 4022). There was no evidence of publication bias.

**Conclusion:**

Although epidemic and pandemic response work may add only a small additional burden, improving mental health through service management and provision of mental health services should be a priority given that baseline rates of poor mental health are already very high. As new studies emerge, they are being added to a living meta-analysis where all analysis code and data have been made freely available: https://osf.io/zs7ne/.

**Electronic supplementary material:**

The online version of this article (10.1007/s00127-020-01990-x) contains supplementary material, which is available to authorized users.

## Introduction

The recent SARS-CoV-2/COVID-19 pandemic has seen an increased demand on clinical staff, who need to treat large numbers of patients, often in newly-purposed wards, with little disease-specific evidence to guide treatment. Many clinical staff have been moved into new roles and may be managing acutely unwell patients using unfamiliar equipment. Stresses caused by high patient mortality rates, staffing shortages, concerns about infecting self or family members, and changing guidance on personal protective equipment can add to work pressure. This has raised concerns about the potential impact on the mental health of epidemic and pandemic responders [1, 2].

Importantly however, high rates of poor mental health are common in clinical staff working in acute medicine generally [3–5] and so estimating the additional impact of epidemic and pandemic response, not solely the extent of poor mental health, is also important in guiding decisions to protect staff mental health. Recently, a meta-analysis by Kisely et al. [6] reported that healthcare workers in direct contact with affected patients had an approximately 1.71–1.74 odds ratio of reporting acute mental health problems compared to healthcare workers not in direct contact with infected patients, and when analysing continuous outcomes, increased scores equivalent to an effect size (standardised mean difference or Hedge’s g) of approximately 0.2–0.4.

Given that the SARS-CoV-2/COVID-19 pandemic is likely to evolve in location, course and severity over time, traditional meta-analyses are only likely to give a snapshot of the current state of the evidence. Living systematic reviews or meta-analyses are a new innovation that allow estimates to be updated as new evidence emerges [7, 8] but have rarely been used to date.

Consequently, we report a rapid review and meta-analysis that forms the basis of a living meta-analysis of the mental health of clinical staff dealing with epidemics and pandemics of high-risk infectious diseases, including studies from coronavirus disease 2019 (COVID-19), Ebola virus disease, H1N1 influenza, severe acute respiratory syndrome (SARS), and middle east respiratory syndrome (MERS) to understand the potential impact on mental health and to inform policy on supporting staff during the current COVID-19 pandemic. The meta-analysis will be updated as new evidence become available and is published on the Open Science Framework.

## Methods

There is no standardised procedure for conducting rapid reviews, although several approaches have been used to reduce the complexity of the review process [9]. We used an iterative rapid review procedure to identify relevant articles and then used the reference lists of already included studies to identify further articles. We searched PubMed, Medline, PsychInfo and Embase for articles including ‘mental health’ or ‘psychosocial’ or ‘emotional’ and ‘staff’ and a number of disease-specific key words (epidemic, epidemics, pandemic, flu, SARS, MERS, COVID-19, Ebola, Marburg, H1N1, H7N6) in the title. We also searched pre-print servers MedRXviv and SSRN for pre-prints relating to COVID-19.

**Fig. 1:**
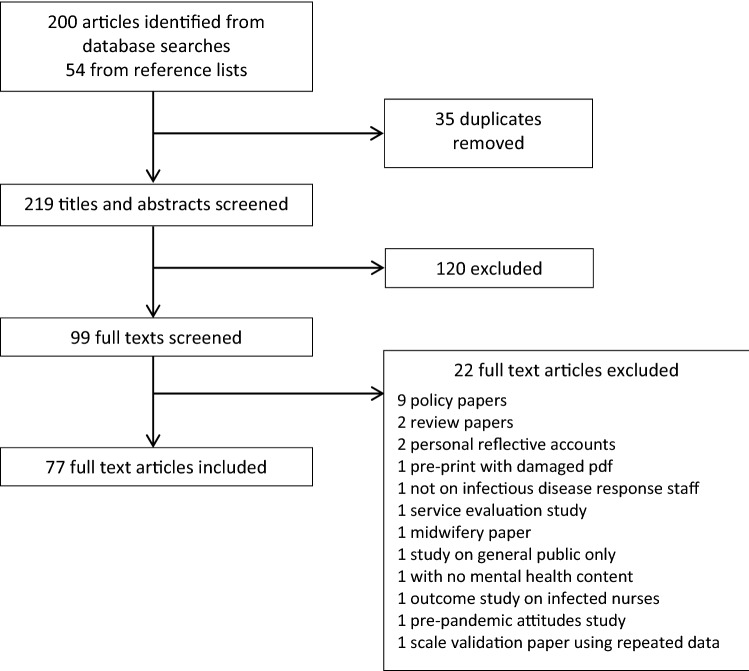
Study screening process

Duplicate articles were removed and titles and abstracts were screened for relevance. The full text of the remaining articles was read and reference lists of these papers were searched for additional relevant articles. These were reviewed for inclusion using the same method as articles found through database searches. Articles were included if they were peer-reviewed articles published in English or Spanish (the languages of the authors) that reported mental health outcomes in clinical staff managing high-risk infectious disease outbreaks. We considered studies on all healthcare staff in all settings as relevant. One article in Chinese with numeric results reported in the English-language abstract was also included. Studies of methodologies including quantitative studies, qualitative studies, and anecdotal accounts, reporting original results were included. Following methods for improving rapid review study selection [10], selection was conducted by the first author and checked by the second author. A flowchart of the review process is presented in (Fig. 1).

The final count of articles included by condition was: COVID-19: 14, Ebola: 11, H1N1: 4, MERS: 6, SARS: 39. No relevant studies on Marburg or H7N6 flu were found. Studies were conducted in China (17), Hong Kong (12), Taiwan (11), Canada (8), Singapore (7), Sierra Leone (4), South Korea (4), Saudi Arabia (3), Germany (1), Greece (1), Israel (1), Japan (1), Liberia (1), Mexico (1), Uganda and the Republic of Congo (1), United States (2), and two studies that recruited aid workers who had worked in various West African countries.

Numeric data from studies reporting (i) above cut-off prevalence or, (ii) means and standard deviations—from validated anxiety, post-traumatic stress disorder (PTSD) and depression symptom scales were extracted by the lead author and checked by the second. Anxiety and depression measures were included on the basis of being validated measures of anxiety or depression, either as the sole focus of a scale or a validated subscale. PTSD symptoms were measured by PTSD-specific scales.

Due to studies using differing cut-offs for determining caseness even when using the same scale, meta-analysis for prevalence was not possible. However, two types of meta-analytic estimates were possible: a comparison of risk ratios for studies that compared above-cut off scoring between high- and low-exposure groups, and meta-analysis for differences in mean scale scores between high and low exposures—both of which were used to help determine the mental health impact of work involving high exposure to infected patients in epidemic and pandemic health emergencies. Individual studies varied in the extent to which cut-off they used for particular scales. When prevalence for several cut-offs was reported (e.g. for mild, moderate, severe), we included the prevalence for the ‘moderate’-level cut-off to select the prevalence likely to reflect a clinically significant problem.

We defined high and low exposures as direct contact with infected patients, or work in wards where direct contact was considered highly likely (e.g. critical care, designated treatment wards, emergency departments) compared to clinicians working without direct contact or work in hospital areas where direct contact was unlikely. A narrative synthesis of risk factors was conducted across all studies regardless of methodology.

As part of the rapid review process, a risk of bias assessment was only conducted for studies included in the meta-analyses using tools to assess risk of bias in cohort studies [11] and case–control studies [12]. The risks of bias assessment evaluations were conducted by one author and checked by the other.

We conducted a power analysis for meta-analysis [13] to determine the minimum number of studies required to detect a statistically significant difference in standardised mean difference between high- and low-exposure groups. We calculated power to detect a small effect size and based on results of the Brooks et al. [14] systematic review of SARS response studies, assumed medium study heterogeneity and an average group size of *N* = 150. This indicated that a minimum study count of 5 was needed. Effect sizes were calculated as standardised mean differences and we used a random effects model to estimate pooled effect sizes. The possibility of publication bias was assessed using and Egger’s test [15]. All analyses were conducted with *R* (version 4.0.2) using the ‘meta’ package (version 4.13.0) and were conducted on a Linux x86_64 platform. All codes and data for the fixed reference analysis reported in this paper are available online in the following archive: https://osf.io/xtecb/.

### Living meta-analysis methods

The living meta-analysis is updated based on the same protocol reported here but only includes studies reporting quantitative data for the purposes of updating the meta-analysis. Literature searches will be repeated at least monthly by the authors. Studies included in the ongoing meta-analysis will be monitored for retractions using the automatic retraction notification function of reference manager Zotero. Reviewers will also monitor the literature and will replace studies included as pre-prints with their final versions when they are published in the peer review literature. We aim to continue with the living meta-analysis as long as it is feasible and will add a notice to the living review if the research team is no longer updating it.

The living meta-analysis has been implemented using Jupyter notebooks which are a type of executable document that allows analysis code and text to be combined in a single document [16]. Following recommendations for research responding to the COVID-19 pandemic [17] all data, and Jupyter notebooks including analysis code and output used in the living meta-analysis have been made freely available at an Open Science Framework archive. This approach also allows other researchers to replicate, modify, or continue the project independently of the authors.

The living meta-analysis is available online at the following address: https://osf.io/zs7ne/.

## Results

The full details of included studies are in Tables S1–3 in the supplementary material and a summary of quantitative and qualitative papers included are shown in Tables 1 and 2 below. All studies included in the living meta-analysis of quantitative studies are included in the data tables published in the online archive.

**Table 1:** Included quantitative studies for the analysis reported in this paper

Study and year	Disease	Location(s)
Alsubaie et al. 2019 [18]	MERS	Saudi Arabia
Austria-Corrales et al. 2011 [19]	H1N1	Mexico
Bai et al. 2004 [20]	SARS	Taiwan
Bukhari et al. 2016 [21]	MERS	Saudi Arabia
Chan and Huak 2004 [22]	SARS	Singapore
Chan et al. 2005 [23]	SARS	Hong Kong
Chang et al. 2006 [24]	SARS	Taiwan
Chen et al. 2005a [25]	SARS	Taiwan
Chen et al. 2006 [26]	SARS	Taiwan
Chong et al. 2004 [27]	SARS	Taiwan
Chua et al. 2004 [28]	SARS	Hong Kong
Chung and Yeung 2020 [29]	COVID-19	Hong Kong
Dai et al. 2020 [30]	COVID-19	China
Fiksenbaum et al. 2006 [31]	SARS	Canada
Goulia et al. 2010 [32]	H1N1	Greece
Grace et al. 2005 [33]	SARS	Canada
Ho et al. 2005 [34]	SARS	Hong Kong
Huang et al. 2020 [35]	COVID-19	China
Huang et al. 2020 [36]	COVID-19	China
Iancu et al. 2005 [37]	SARS	Israel
Ji et al. 2017 [38]	Ebola	Sierra Leone
Jung et al. 2020 [39]	MERS	South Korea
Khalid et al. 2016 [40]	MERS	Saudi Arabia
Koh et al. 2005 [41]	SARS	Singapore
Lai et al. 2020 [42]	COVID-19	China
Lancee et al. 2008 [43]	SARS	Canada
Lee et al. 2018 [44]	MERS	South Korea
Lee et al. 2005 [45]	SARS	Taiwan
Lehmann et al. 2016 [46]	Ebola	Germany
Li et al. 2015 [47]	Ebola	Liberia
Li et al. 2020 [48]	COVID-19	China
Liang et al. 2020 [49]	COVID-19	China
Lin et al. 2007 [50]	SARS	Taiwan
Liu et al. 2020 [51]	COVID-19	China
Liu et al. 2012 [52]	SARS	China
Liu et al. 2020 [53]	COVID-19	China
Lung et al. 2009 [54]	SARS	Taiwan
Marjanovic et al. 2007 [55]	SARS	Canada
Matsuishi et al. 2012 [56]	H1N1	Japan
Maunder et al. 2006 [57]	SARS	Canada
McAlonan et al. 2007 [58]	SARS	Hong Kong
Nickell et al. 2004 [59]	SARS	Canada
Oh et al. 2017 [60]	MERS	South Korea
Park et al. 2018 [61]	MERS	South Korea
Phua et al. 2008 [62]	SARS	Singapore
Poon et al. 2004 [63]	SARS	Hong Kong
Qi et al. 2020 [64]	COVID-19	China
Sim et al. 2004 [65]	SARS	Singapore
Styra et al. 2008 [66]	SARS	Canada
Su et al. 2007 [67]	SARS	Taiwan
Sun et al. 2020 [68]	COVID-19	China
Tam et al. 2004 [69]	SARS	Hong Kong
Tan et al. 2020 [70]	COVID-19	Singapore
Tham et al. 2004 [71]	SARS	Singapore
von Strauss et al. 2017 [72]	Ebola	Various in West Africa
Waterman et al. 2018 [73]	Ebola	Sierra Leone
Wong et al. 2005 [74]	SARS	Hong Kong
Wong et al. 2004 [75]	SARS	Hong Kong
Wu et al. 2008 [76]	SARS	China
Wu et al. 2009 [77]	SARS	China
Xiao et al. 2020 [78]	COVID-19	China
Xu et al. 2020 [79]	COVID-19	China
Zhang et al. 2020 [80]	COVID-19	China
Zhu et al. 2020 [81]	COVID-19	China

**Table 2:** Included qualitative studies for the analysis reported in this paper

Study and year	Disease	Location(s)
Chiang et al. 2007 [82]	SARS	Taiwan
Chung et al. 2005 [83]	SARS	Hong Kong
Cunningham et al. 2017 [84]	Ebola	Various in West Africa
Hewlett and Hewlett 2005 [85]	Ebola	Uganda, Republic of Congo
McMahon et al. 2016 [86]	Ebola	Sierra Leone
Meyer et al. 2018 [87]	Ebola	United States
Raven et al. 2018 [88]	Ebola	Sierra Leone
Shih et al. 2009 [89]	SARS	Taiwan
Smith et al. 2017 [90]	Ebola	United States
Wong et al. 2012 [91]	H1N1	Hong Kong

Meta-analytic estimates of impact of working in high exposure roles are presented in Tables 3 and 4. The forest plots for each analysis reported in this paper are reported in the supplementary material. Forest plots for all quantitative studies included in the living meta-analysis are published in the Jupyter notebooks on the online archive.

**Table 3:** Meta-analytic estimates for differences in prevalence of mental health problems between high-exposure and low-exposure work calculated as risk ratios (RR) with study heterogeneity (*I*^2^)

	RR	RR 95% CI	Studies	Total *N*	*I*^2^ (%)
Anxiety	1.30	0.87–1.93	4	12,473	88
PTSD symptoms	1.16	0.75–1.78	7	6604	79
Depression	1.50	0.57–3.95	5	12,224	81

**Table 4:** Meta-analytic estimates for differences in mean scores of symptom measure scales between high-exposure and low-exposure work calculated as standardised mean difference (SMD) with study heterogeneity (*I*^2^)

	SMD	SMD 95% CI	Studies	Total *N*	*I*^2^ (%)
Anxiety	0.16	0.02–0.31	11	6493	79
PTSD symptoms	0.20	0.01–0.40	11	5122	87
Depression	0.13	− 0.04–0.31	8	4022	81

The confidence intervals for estimates of risk ratio crossed 1 (signifying equal ratio and therefore no estimate difference between high- and low-exposure groups) for all differences based on prevalence of above cut-off scoring. The confidence intervals for standardised mean difference crossed zero only for depression. However, study heterogeneity was larger than we assumed during the power calculation and there were the fewest number of studies included for the depression meta-analysis (*k* = 8), meaning it was likely under-powered to detect small effects.

For those estimates where we had the minimum number of 10 studies [92] to test for publication bias (i.e. the estimates of SMDs for anxiety and PTSD symptoms), Egger’s test indicated no evidence for publication bias (anxiety: *p* = 0.614; PTSD symptoms: *p* = 0.212).

The assessment of individual study risk of bias (see supplementary materials) indicated that included studies showed moderate to high risk of bias.

### Risk factors, protective factors and additional work stresses

Although it was not possible to examine risk and protective factors meta-analytically, a narrative synthesis was conducted to identify likely candidates.

Nurses typically reported higher levels of symptoms and distress than doctors [32, 35, 42, 56, 59, 63, 71, 81, 93] with a few studies reporting no difference [30, 57, 68] and one study reporting higher rates in doctors [22].

Several studies noted that seeing colleagues infected was a particular source of distress [30, 40, 88]. Furthermore, being quarantined after infection was reported as a predictor of psychological distress and poor mental health [20, 30, 31, 52, 55, 66, 76, 77] which was also reflected in one qualitative study [94] although three studies found no negative impact of quarantine [27, 66, 95].

Notably, numerous studies reported that clinical staff dealing with high-risk infectious disease experienced stigma from friends, family and the public [20, 32, 33, 57, 59, 61, 63, 66, 72, 85–87, 90, 96, 97] and perceived stigma was found to be a predictor of poor mental health in the three studies that looked at this association statistically [57, 61, 97].

Three studies found that clinical staff who were conscripted, were not willing, or had not volunteered for high-exposure roles reported particularly poor mental health outcomes [26, 30, 69, 98].

Although small in number, longitudinal studies suggested that symptoms of poor mental health tend to peak early during outbreaks but resolve for the majority of responders as time goes on [5, 26]. This pattern of initially high levels of anxiety and distress that reduce over time was also reflected in some of the qualitative studies [83, 89]. One cross-sectional study conducted at two time points and sampling from the same clinical teams found high levels of self-reported poor mental health for high-exposure workers 1 year after high-exposure work [57] although a study on a subsample of participants using a structured interview assessment found the incidence of new onset mental health problems was essentially no different to that found in the general population [43].

In terms of protective factors that reduced the chance of poor mental health or psychological distress, social support, team cohesion or organisational support were identified by numerous studies [22, 24, 31, 40, 45, 55, 68, 72, 78, 81]. This theme was reflected in several qualitative studies [87, 88].

Furthermore, the use, availability, training with, and faith in, infection prevention measures were identified as reducing distress [28, 40, 45, 45, 55, 57, 59, 65, 81]

A sense of professional duty and altruistic acceptance of risk was found to be a protective factor in several studies [32, 52, 60, 77] which was a theme that was strongly reflected in qualitative studies [83, 85, 88, 91] although one study found accepting the risk of SARS infection as part of the job was not associated with reduced psychopathology [97].

Notably, all studies that asked about positive aspects of working in epidemic and pandemic response reported that participants described several factors related development as an individual and a team, particularly in terms of learning new skills and medical knowledge, better adherence to medical procedures, and closer team working and collegiality [28, 33, 59].

### Role of formal psychological support services

Although a recent anecdotal report noted clinicians did not find mental health support particularly useful during COVID-19 response [1], several studies found that participants reported formal psychological services to be a useful source of support [32, 45, 72, 87, 90]. One study specifically asked whether staff needed ‘psychological treatment’ and 8.6% of healthcare workers dealing with COVID-19 reported they did [93]. Conversely, however, Chung and Yeung [29] reported that only 2% of staff responding to COVID-19 requested psychological support and all “were reassured after a single phone contact by the psychiatric nurse” although this was a small study with just 69 participants.

Notably, two large COVID-19 studies suggest that the staff who are most in need of psychological support are the least likely to request or receive it. One study found that high exposure staff were likely to say they needed psychological treatment at half the rate of low exposure staff despite reporting higher levels of psychopathology [93]. In another, clinicians with mental health problems were less likely to receive psychological support than clinicians without [53].

## Discussion

To estimate the impact of high-exposure work in epidemic and pandemic health emergencies, we completed a rapid review and meta-analysis of studies reporting on the mental health of clinical staff working in high-risk epidemic and pandemic health emergencies, including studies from the recent COVID-19 pandemic. These comparisons suggest that the impact of epidemic and pandemic response work on mental health is likely to be small, but in the case of meta-analytic assessment of mean scale scores, it is statistically detectable. However, it is worth noting that this small additional burden is in addition to already high levels of poor mental health that are common in acute medical staff. A narrative synthesis of potential risk factors for poor mental health in epidemic and pandemic response identified being a nurse, experiencing stigma from others, seeing colleagues infected, and being personally quarantined as predictors of worse outcomes. Protective factors included social and occupational support, effectiveness and faith in infection control measures, a sense of professional duty and altruistic acceptance of risk. Formal psychological services were identified as a valuable form of support.

It is worth noting some of the shortcomings of the evidence used to inform this review. More studies reported results from the analysis of mental health measures than reported sufficient detail (prevalence, mean scores etc.) to allow them to be included in the numeric assessment of results in this review. In terms of the assessment of individual studies, there was at least a moderate risk of bias although there was no suggestion of publication bias in the literature where this could be tested statistically. Furthermore, studies almost exclusively used self-report measures rather than structured interview assessments. Occasionally, some studies reported two categorisations that would potentially count as ‘high risk’—for example, ‘working in isolation wards’ and ‘working directly with infected patients’. In these situations, we chose what seemed like the most likely to reflect ‘direct contact’ and have opted for transparency through making our decisions clear in the open datasets. We also note potential shortcomings of the methods employed here. The rapid review approach is necessarily less thorough than a full systematic review and it is possible that the search strategy employed here may not exhaustively identify all relevant articles. However, we also note that the living meta-analysis approach has the potential to allow us to include ‘missed’ articles as they are identified. Additionally, there may be data from articles published in languages not accessible to the research team and this may reduce the international coverage of the analysis.

Notably, the results reported here largely echo those reported in a recent rapid-review and meta-analysis by Kisely et al. [6], although some differences are worth highlighting. In contrast to the analysis reported here, Kisely et al. created meta-analytic summaries of: (i) acute/post-traumatic stress, and (ii) psychological distress—that combined measures of burnout, depression, and general psychopathology. However, a similar pattern emerges. The effect sizes were relatively small and somewhat more pronounced for psychological distress, which is more likely to reflect acute reactions rather than longer-term mental health problems. One benefit of our living meta-analysis approach is that we will be able to see whether this pattern of results remains intact. Indeed, given the varying course of the COVID-19 pandemic it is possible that we may see increases in mental health problems in clinical staff if the pandemic endures for a far greater time than comparable outbreaks, or differs in severity between countries, where national differences may arise.

The results of this review and meta-analysis raise several issues with regard to provide ongoing and long-term support for clinical staff responding to such health emergencies. One is the extent to which epidemic and pandemic response is uniquely ‘traumatising’ and might lead to high levels of posttraumatic stress disorder, thereby implying that a trauma-focus for staff support should be the dominant approach to addressing outcomes of poor mental health. With regard to the published studies, although there are seemingly high rates of staff who score above cut-off on measures of PTSD symptoms, interpretation of results is not straightforward. Cut-off values used for defining a positive ‘case’ varied considerably even between studies using the same measure. The most widely used scale in these studies is the Impact of Events Scale-Revised for which a cut-off of 33–34 has been found to be the most predictive of diagnosable PTSD [99, 100] and yet most studies use a cut-off of considerably less. This suggests that an important proportion of those reported under the prevalence figures are likely to have transitory, sub-syndromal PTSD symptoms, or non-specific distress, that may be a risk for PTSD but are unlikely to reach the level of a diagnosable case. Indeed, the prevalences reported here are comparable to prevalences found in clinical staff more widely. For example, the reported prevalence of > 33 scoring on the IES-R is 15% in acute medical staff [101], 16% in surgical trainees [102], and 17% in cancer physicians [103]. Studies included in this review using similar IES-R cut-offs to these general medicine studies tended to report lower prevalence rates with only one study [81] reporting higher.

Furthermore, studies often did not differentiate between PTSD symptoms arising from pandemic and epidemic response work and those from other events, meaning it is not clear to what extent epidemic or pandemic response work was the key causal factor. Finally, symptoms were almost exclusively measured by self-report measures which are known to inflate the rate of true cases [104]. Indeed, high rates on self-report measures but low rates on structured interview assessments have been found in SARS responders [43].

This suggests that although there is potential for trauma and this should be included in considerations for staff support, it is currently not clear at the moment that PTSD is an outcome particularly associated with epidemic and pandemic response meaning support efforts should be ‘trauma-ready’ rather than ‘trauma-focused’. However, we note here the importance of contextual factors. The capacity of the healthcare system, and indeed the population, are key contextual factors in determining the impact of the medical response on the staff responsible for delivering it. Although there is no strong evidence from the data we report here for the differential impact of managing specific infectious diseases, indicators, such as mortality and morbidity, infectiousness, and social perception, should be monitored as potential risk factors for poor mental health.

Indeed, some of these contextual factors were reflected in the risk and protective factors identified in the review. These chime with previous work on epidemic response work [14] and the wider literature on mental health outcomes in high-risk work [105] and suggest similar measures to support staff, namely promoting good leadership and team cohesion, maintaining high standards of infection control and training. In addition, this review highlights that additional attention should be given to nurses, those affected by seeing colleagues infected, where staff are quarantined after being infected, and where individuals experience stigma from others.

It is worth highlighting that formal psychological support was considered useful by clinical staff but that it was least requested and less frequently received by those with higher levels of mental health difficulties. Although voluntary engagement with mental health services is considered the ideal model to avoid potential iatrogenic effects [106], particular attention should be paid to pathways to formal support to make these as accessible as possible for those who need them. This is particularly important as staff working in acute medicine may already have high levels of poor mental health and access to effective treatment during health emergencies should be a priority. Considering there are no evidence-based interventions that have been shown to reduce poor mental health outcomes in those responding to healthcare emergencies, we agree with Greenberg et al. [2] that structural and systemic factors are likely to be key—namely, good leadership, the effective use and availability of protective measures, promoting team cohesion, and quality aftercare. Nevertheless, additional research on how epidemic response work impacts on mental health and on the most effective provision and timing of effective interventions is clearly a research priority.

### Role of living meta-analysis

The authors will be updating the living meta-analysis as new data becomes available to give the most up-to-date estimate of potential impact on the mental health of clinical staff. However, we realise that meta-analyses involve decisions at the inclusion, data extraction and analysis stages that may differ by the objectives of the researchers. For this reason, we have also made the data and the analysis code fully open to allow for full transparency, but also to facilitate others re-running the analysis with alternative decisions to examine the impact on outcome. We suggest that prioritising transparency and re-use of previous work is important in time critical health emergencies and we encourage other researchers to adopt this approach.

### Priorities for research

The majority of studies are cross-sectional measuring mental health in staff working in epidemic and pandemic response. These studies typically have large sample sizes and good response rates. However, there remains a need for: (i) standardisation of measures, reporting, and criteria for prevalence; (ii) adequately sampled case–control studies that compare epidemic and pandemic response staff to control groups of other staff in acute medicine; and (iii) longitudinal studies to examine the course of psychological distress over time. We also note that (i) would effectively be solved if open data were available from the relevant studies, and we strongly encourage researchers to make these data available, particularly when researching infectious disease health emergencies.

## Electronic supplementary material

Below is the link to the electronic supplementary material.

Supplementary file1 (DOCX 1819 KB)

Supplementary file2 (DOCX 119 KB)

Supplementary file3 (DOCX 23 KB)
