# Supplementary material for: Mental health of clinical staff working in high-risk epidemic and pandemic health emergencies a rapid review of the evidence and living meta-analysis
Source: Soc Psychiatry Psychiatr Epidemiol. 2020 Nov 27;56(1):1–11. doi: 10.1007/s00127-020-01990-x (PMC7691696; doi:10.1007/s00127-020-01990-x)
Supplement: Supplementary file 1 — Supplementary file1 (DOCX 1819 KB) [file 127_2020_1990_MOESM1_ESM.docx]

**Figure S1. Forest plot of risk ratio meta-analysis for anxiety symptoms**

**
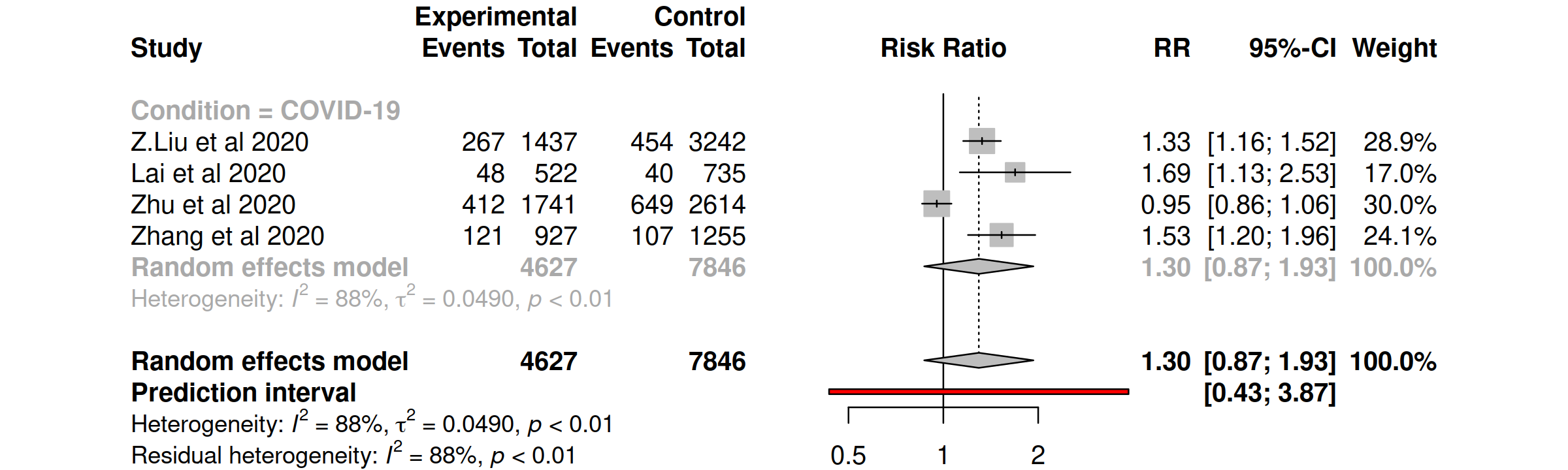
**

**Figure S2. Forest plot of risk ratio meta-analysis for PTSD symptoms**

**
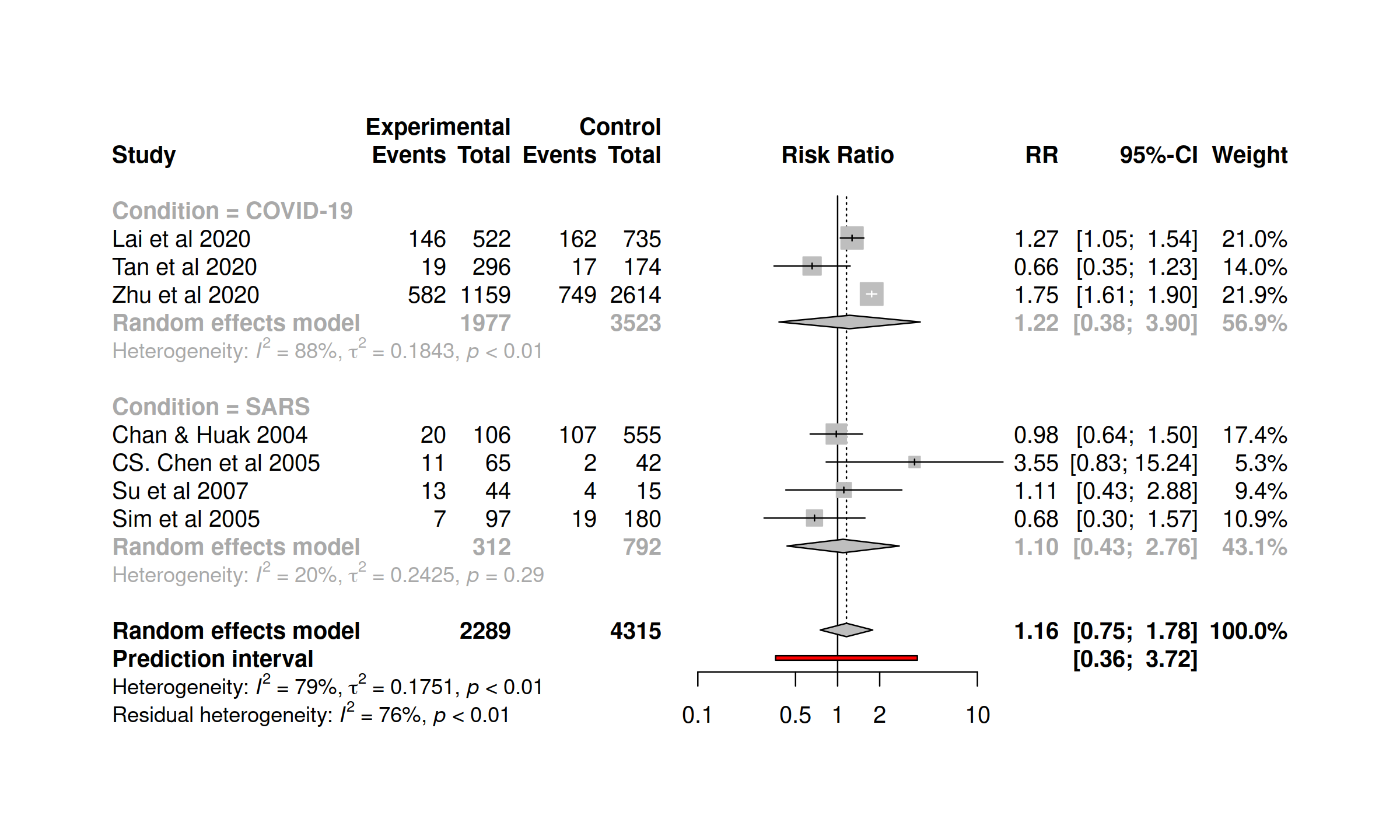
**

**Figure S3. Forest plot of risk ratio meta-analysis for depression symptoms**

**
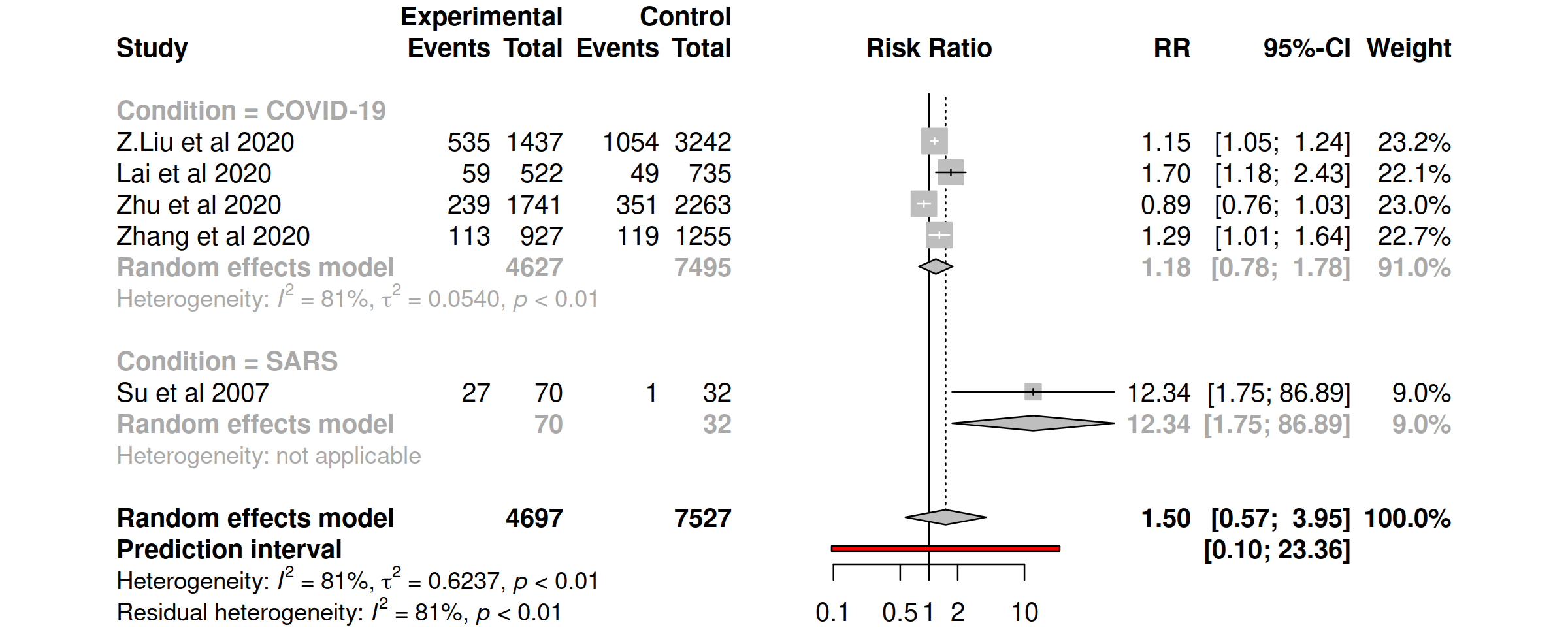
**

**Figure S4. Forest plot of standardized mean difference (Hedge’s g) meta-analysis for anxiety symptoms**

**
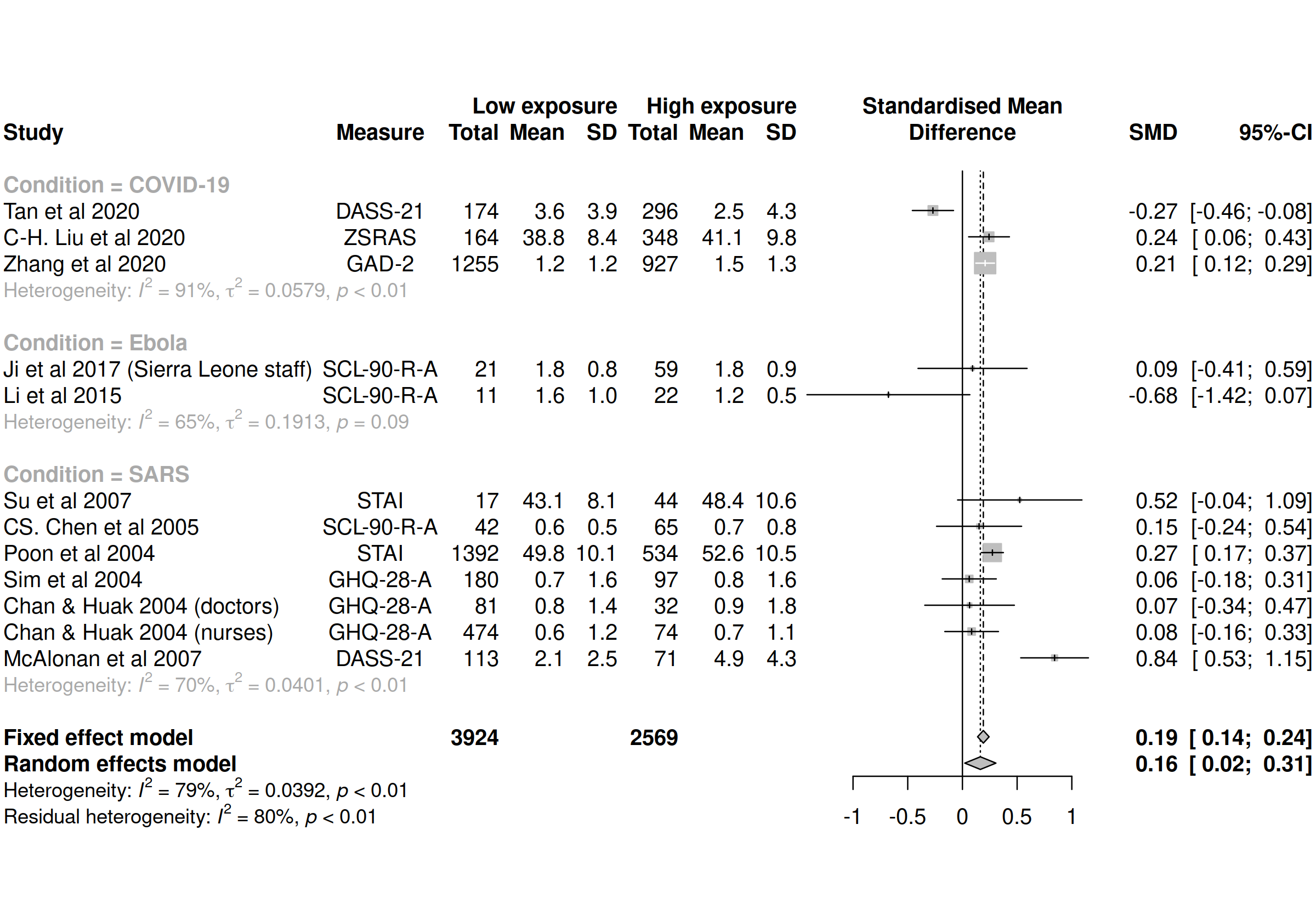
**

**Figure S5. Forest plot of standardized mean difference (Hedge’s g) meta-analysis for PTSD symptoms**

**
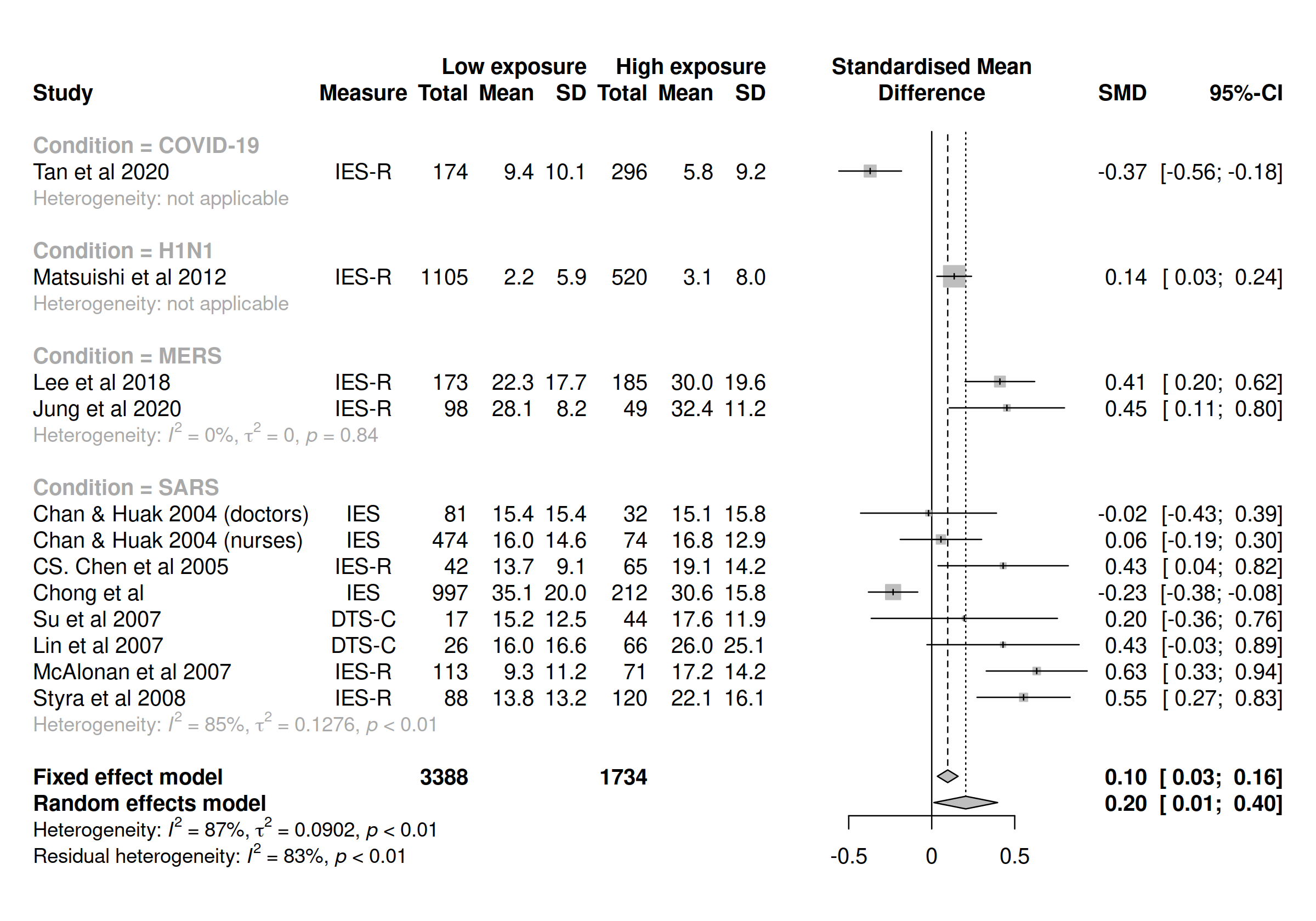
**

**Figure S6. Forest plot of standardized mean difference (Hedge’s g) meta-analysis for depression symptoms**

**
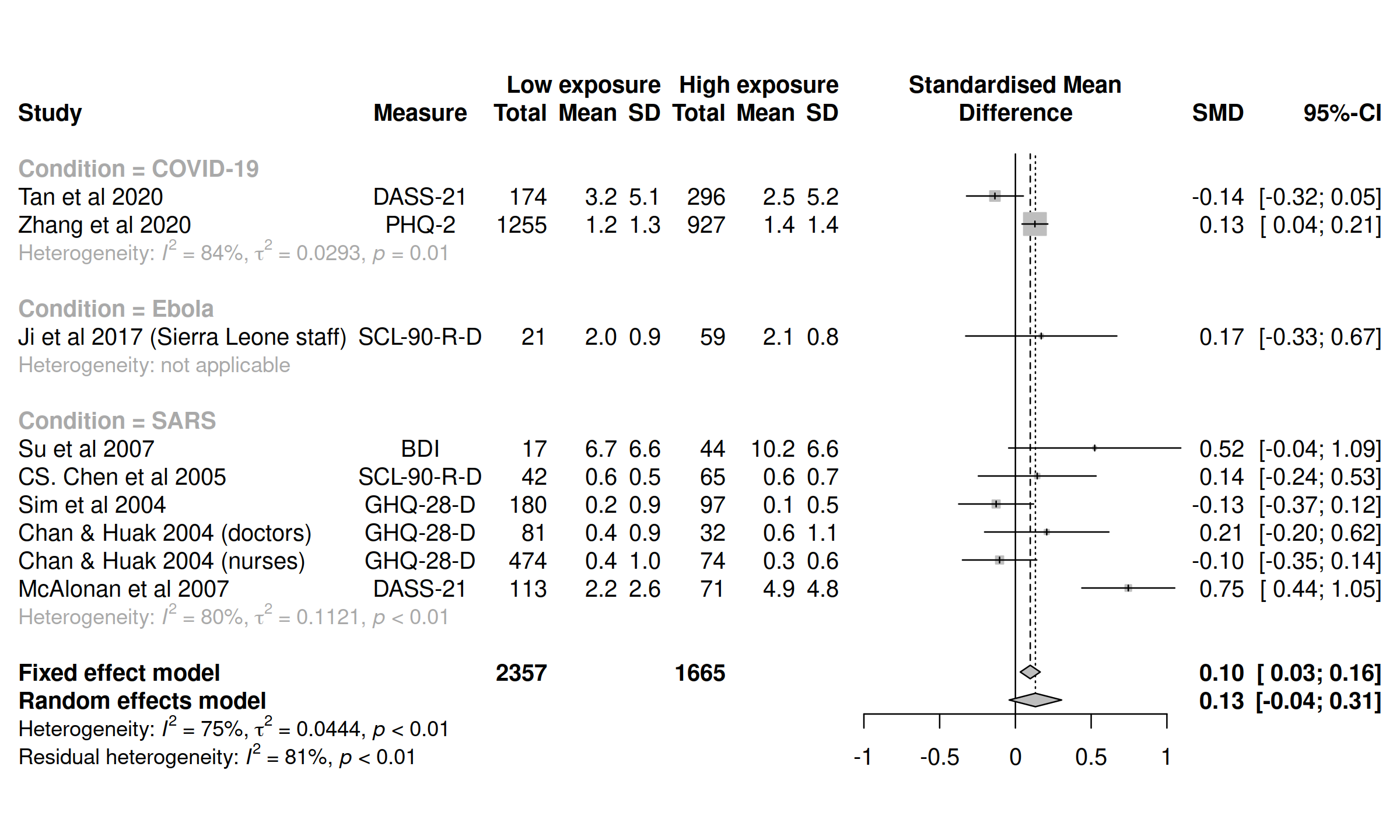
**

**Table S1. All studies that measured anxiety prevalence**

|  |  |  |  |  |  | *Prevalence* | | |  |  |
| --- | --- | --- | --- | --- | --- | --- | --- | --- | --- | --- |
| *Study* | *Condition* | *Measure* | *Cut-off used* | *Sample* |  | *Overall* | *High exposure* | *Low exposure* |  | *Timing* |
| Xiao et al 2020 [1] | COVID-19 | ZSRAS | Not used | HE only |  | Not given | Not given | Not given |  | During |
| C-H. Liu et al 2020 [2] | COVID-19 | ZSRAS | >50 | HE and LE |  | 12.5% | Not given | Not given |  | During |
| Z. Liu et al 2020 [3] | COVID-19 | ZSRAS | >49 | HE and LE |  | 16.0% | 18.6% | 14.0% |  | During |
| J. Huang et al 2020 [4] | COVID-19 | ZSRAS | Not given | HE only |  | Not relevant | 23.04% | Not relevant |  | During |
| Liang et al 2020 [5] | COVID-19 | ZSRAS | Not used | HE and LE |  | Not given | Not given | Not given |  | During |
| Lai et al 2020 [6] | COVID-19 | GAD-7 | >4 | HE and LE |  | 44.6% | 51.3% | 39.4% |  | During |
| Zhu et al 2020[7] | COVID-19 | GAD-7 | >7 | HE and LE |  | 24.36% | 23.66% | 24.83% |  | During |
| Sun et al 2020 [8] | COVID-19 | SCL-90-A | Not used | HE and LE |  | Not given | Not given | Not given |  | During |
| Tan et al 2020 [9] | COVID-19 | DASS-21 | >7 | HE and LE |  | 14.5% | 10.8% | 20.7% |  | During |
| Zhang et al 2020 [10] | COVID-19 | GAD-2 | >2 | HE and LE |  | 10.4% | 13.0% | 8.5% |  | During |
| Ji et al 2017 [11] | Ebola | SCL-90-R-A | Not used | HE and LE |  | Not given | Not given | Not given |  | During |
| McAlonan et al 2005 [12] | SARS | DASS-21-A | Not given | HE and LE |  | Not given | Not given | Not given |  | During & 1 year |
| R. Chen et al 2006 [13] | SARS | ZSRAS | >59* | HE Only |  | Not relevant | Not given | Not relevant |  | During, T1 |
| Iancu et al 2005 [14] | SARS | MSAS | Not used | Undifferentiated |  | Not given | Not given | Not given |  | During |
| C.S. Chen et al 2005 [15] | SARS | SCL-90-R-A | Not used | HE and LE |  | Not given | Not given | Not given |  | During |
| Poon et al 2004 [16] | SARS | STAI | Not used | HE and LE |  | Not given | Not given | Not given |  | During |

ZSRAS = Zung Self-rating Anxiety Scale; MSAS = Modified Spielberger Anxiety Scale; SCL-90-A = Symptom Checklist 90 Anxiety Subscale; SCL-90-R-A = Symptom Checklist 90 Revised Anxiety Subscale; DASS-21 = Depression, Anxiety and Stress Scale

**Table S1.** All studies measuring anxiety symptom prevalence for healthcare responders for high risk epidemic and pandemic disease outbreak using validated scales

**Table S2. All studies that measured PTSD symptom prevalence**

|  |  |  |  |  |  | *Prevalence* | | |  |  |
| --- | --- | --- | --- | --- | --- | --- | --- | --- | --- | --- |
| *Study* | *Condition* | *Measure* | *Cut-off used* | *Sample* |  | *Overall* | *High exposure* | *Low exposure* |  | *Timing* |
| J. Huang et al 2020 [4] | COVID-19 | PTSD-SS | Not given | HE only |  | Not relevant | 27.39% | Not relevant |  | During |
| Lai et al 2020 [6] | COVID-19 | IES-R | >8 | HE and LE |  | 71.5% | 76.0% | 68.0% |  | During |
| Zhu et al 2020[7] | COVID-19 | IES-R | >33 | HE and LE |  | 30.56% | 33.43% | 28.65% |  | During |
| Xiao et al 2020 [1] | COVID-19 | SASR | Not given | HE only |  | Not given | Not given | Not given |  | During |
| Tan et al [9] | COVID-19 | IES-R | >24 | HE and LE |  | 7.7% | 5.7% | 10.9% |  | During |
| Matsuishi et al 2020 [17] | H1N1 | IES-R | Not given | HE and LE |  | Not given | Not given | Not given |  | During |
| Lee et al 2018 [18] | MERS | IES-R | >24 | HE and LE |  | 51.5% | Not given | Not given |  | During |
| Bukhari et al 2016 [19] | MERS | IES | Idiosyncratic | HE and LE |  | Not given | Not given | Not given |  | During |
| Chan &Huak 2004 (doctors)[20] | SARS | IES | >29 | HE and LE |  | Not given | 18.8% | 19.8% |  | During |
| Chan & Huak 2004 (nurses)[20] | SARS | IES | >29 | HE and LE |  | Not given | 19.4% | 19.5% |  |  |
| Chong et al 2004 [21] | SARS | IES | Not given | HE and LE |  | Not given | Not given | Not given |  | During |
| Koh et al 2005 [22] | SARS | IES | >16 | HE and LE |  | Not given | Not given | Not given |  | During |
| C.S. Chen et al 2005[15] | SARS | IES-R | >36 | HE and LE |  | 11% | 17% | 2% |  | During |
| Ho et al 2005 [23] | SARS | CIES-R | Not given |  |  | Not given | Not given | Not given |  | During |
| Su et al 2007 [24] | SARS | DTS-C | >22 | HE and LE |  | 28.43% | 32.86% | 18.75% |  | During |
| Sim et al 2004 [25] | SARS | IES | Idiosyncratic | HE and LE |  | 9.4% | 7.2% | 10.6% |  | During |
| Styra et al 2008 [26] | SARS | IES-R | >19 | HE and LE |  | Not given | Not given | Not given |  | During |
| Lin et al 2007 [27] | SARS | DTS-C | >40 | HE and LE |  | 19.3% | 21.7% | 13.0% |  | During |
| Tham et al 2004 / Phua et al 2005 [28, 29] | SARS | IES | >25 | Undifferentiated |  | 17.7% | Not relevant | Not relevant |  | 6 months after |
| Maunder et al 2006 [30] | SARS | IES | >25 | HE and LE |  | Not given | 13.8% | 8.4% |  | 1-2 years after |
| Lancee et al 2008 [31] | SARS | CAPS | DX | HE only |  | Not relevant | 2% | Not relevant |  | 1-2 years after |
| McAlonan et al 2005 [12] | SARS | IES-R | Not given | HE and LE |  | Not given | Not given | Not given |  | During & 1 year |
| Wu et al 2008/2009 [32][33] | SARS | IES-R | >19 | HE and LE |  | 10.1% | Not given | Not given |  | 3 years after |

PTSD-SS = PTSD Self Report Scale; IES = Impact of Events Scale; IES-R = Impact of Events Scale – Revised; SASR = Stanford Acute Stress Reaction; CIES-R = Chinese Impact of Event Scale-Revised; Chinese version of the Davidson Trauma Scale (DTS-C); CAPS = Clinician Administered PTSD Scale

**Table 2.** All studies measuring PTSD symptom prevalence for healthcare responders for high risk epidemic and pandemic disease outbreak using validated measures

**Table S3. All studies that measured depression prevalence**

|  |  |  |  |  |  | *Prevalence* | | |  |  |
| --- | --- | --- | --- | --- | --- | --- | --- | --- | --- | --- |
| *Study* | *Condition* | *Measure* | *Cut-off used* | *Sample* |  | *Overall* | *High exposure* | *Low exposure* |  | *Timing* |
| Zhu et al 2020 [7] | COVID-19 | PHQ-9 | >9 | HE and LE |  | 13.55% | 13.73% | 13.43% |  | During |
| Lai et al 2020 [6] | COVID-19 | PHQ-9 | >4 | HE and LE |  | 50.4% | 58.4% | 44.6% |  | During |
| Chung & Yeung 2020 [34] | COVID-19 | PHQ-9 | >9 | Undifferentiated |  | 34.8% | Not given | Not given |  | During |
| Liang et al 2020 [5] | COVID-19 | ZSRAD | Not used | HE and LE |  | Not given | Not given | Not given |  | During |
| Z. Liu et al 2020 [3] | COVID-19 | ZSRAD | >49 | HE and LE |  | 34.6% | 37.2% | 32.5% |  | During |
| Tan et al [9] | COVID-19 | DASS-21 | >9 | HE and LE |  | 8.9% | 8.1% | 10.3% |  | During |
| Zhang et al 2020 [10] | COVID-19 | PHQ-2 | >2 | HE and LE |  | 10.6% | 12.2% | 9.5% |  | During |
| R. Chen et al 2006 [13] | SARS | ZSRAD | >59* | HE Only |  | Not given | Not given | Not given |  | During, T1 |
| Su et al 2007 [24] | SARS | BDI | >9 | HE and LE |  | 27.45% | 38.57% | 3.13% |  | During |
| McAlonan et al 2005 [12] | SARS | DASS-21 | Not given | HE and LE |  | Not given | Not given | Not given |  | During & 1 year |
| Wu et al 2008[32] | SARS | CES-D | >15 | HE and LE |  | 22.8% | Not given | Not given |  | 3 years after |

PHQ-9 = Patient Health Questionnaire 9; DASS-21 = Depression, Anxiety and Stress Scale; ZSRAD = Zung Self-rating Depression Scale; BDI = Beck Depression Inventory

**Table 3.** All studies measuring depression symptoms prevalence for healthcare responders for high risk epidemic and pandemic disease outbreak using validated measures

**Table S4. Anxiety averages**

|  |  |  |  |  |  | *Average (SD if mean)* | | |  |  |
| --- | --- | --- | --- | --- | --- | --- | --- | --- | --- | --- |
| *Study* | *Condition* | *Measure* | *Summary stat* | *Sample* |  | *Overall* | *High exposure* | *Low exposure* |  | *Timing* |
| Xiao et al 2020 [1] | COVID-19 | ZSRAS | Mean | HE only |  | Not relevant | 55.26 (14.18) | Not relevant |  | During |
| C-H. Liu et al 2020 [2] | COVID-19 | ZSRAS | Mean | HE and LE |  | 39.56 (8.91) | 41.11 (9.79) | 38.83 (8.38) |  | During |
| Z. Liu et al 2020[3] | COVID-19 | ZSRAS | Not given | HE and LE |  | Not given | Not given | Not given |  | During |
| J. Huang et al 2020 [4] | COVID-19 | ZSRAS | Mean | HE only |  | 42.91 (10.89) | Not given | Not given |  | During |
| Liang et al 2020 [5] | COVID-19 | ZSRAS | Mean | HE and LE |  | Not given | 28.09 (4.98) | 29.36 (4.46) |  | During |
| Lai et al 2020 [6] | COVID-19 | GAD-7 | Median | HE and LE |  | 4.0 | 5.0 | 3.0 |  | During |
| Zhu et al 2020[7] | COVID-19 | GAD-7 | Not given | HE and LE |  | Not given | Not given | Not given |  | During |
| Sun et al 2020 [8] | COVID-19 | SCL-90-A | Mean | HE and LE |  | 1.50 (0.79) | Not given | Not given |  | During |
| Tan et al [9] | COVID-19 | DASS-21 | Mean | HE and LE |  | Not given | 2.45 (4.28) | 3.57 (3.91) |  | During |
| Zhang et al 2020 [10] | COVID-19 | GAD-2 | Mean | HE and LE |  | 1.36 (1.25) | 1.51 (1.28) | 1.25 (1.23) |  | During |
| Ji et al 2017 [11] (Chinese staff) | Ebola | SCL-90-R-A | Mean | HE only |  | Not relevant | 1.83 (0.23) | Not relevant |  | During |
| Ji et al 2017 [11] (Sierra Leone staff) | Ebola | SCL-90-R-A | Mean | HE only |  | Not relevant | 1.84 (0.89) | 1.76 (0.75) |  | During |
| Li et al 2015 [35] | Ebola | SCL-90-R-A | Mean | HE and LE |  | 0.27 (0.42) | 1.16 (0.52) | 1.65 (0.99) |  | During |
| McAlonan et al 2005 [12] | SARS | DASS-21 | Mean | HE and LE |  | Not given | 4.9 (4.3) | 2.1 (2.5) |  | During & 1 year |
| R. Chen et al 2006 [13] | SARS | ZSRAS | Mean | HE only |  | Not relevant | 61 (12.62) | Not relevant |  | During, T1 |
| Su et al 2007 [24] | SARS | STAI | Mean | HE and LE |  | Not given | 48.4 (10.6) | 43.1 (8.1) |  | During |
| Iancu et al 2005 [14] | SARS | MSAS | Mean | Undifferentiated |  | 13.5 (3.5) | Not relevant | Not relevant |  | During |
| C.S. Chen et al 2005 [15] | SARS | SCL-90-R-A | Mean | HE and LE |  | 0.54 (0.54) | 0.67 (0.75) | 0.57 (0.46) |  | During |
| Poon et al 2004 [16] | SARS | STAI | Mean | HE and LE |  | Not given | 52.6 (10.5) | 49.8 (10.1) |  | During |
| Chan & Huak 2004 (doctors) [20] | SARS | GHQ-28 A | Mean | HE and LE |  | Not given | 0.9 (1.8) | 0.8 (1.4) |  | During |
| Chan & Huak 2004 (nurses) [20] | SARS | GHQ-28 A | Mean | HE and LE |  | Not given | 0.7 (1.1) | 0.6 (1.2) |  | During |
| Sim et al 2004 [25] | SARS | GHQ-28 A | Mean | HE and LE |  | Not given | 0.8 (1.6) | 0.7 (1.6) |  | During |

ZSRAS = Zung Self-rating Anxiety Scale; MSAS = Modified Spielberger Anxiety Scale; SCL-90-A = Symptom Checklist 90 Anxiety Subscale; SCL-90-R-A = Symptom Checklist 90 Revised Anxiety Subscale; STAI = SpielbergerTrait Anxiety Inventory.

**Table S4.** All studies reporting averages of validated anxiety symptom scales for healthcare responders for high risk epidemic and pandemic disease outbreaks

**Table S5. PTSD symptom averages**

|  |  |  |  |  |  | *Average (SD if mean)* | | |  |  |
| --- | --- | --- | --- | --- | --- | --- | --- | --- | --- | --- |
| *Study* | *Condition* | *Measure* | *Summary stat* | *Sample* |  | *Overall* | *High exposure* | *Low exposure* |  | *Timing* |
| J. Huang et al 2020 [4] | COVID-19 | PTSD-SS | Mean | HE only |  | 42.92 (17.88) | Not given | Not given |  | During |
| Lai et al 2020 [6] | COVID-19 | IES-R | Median | HE and LE |  | 20 | 22.5 | 17.0 |  | During |
| Zhu et al 2020[7] | COVID-19 | IES-R | Not given | HE and LE |  | Not given | Not given | Not given |  | During |
| Xiao et al 2020 [1] | COVID-19 | SASR | Mean | HE only |  | Not relevant | 77.59 (29.53) | Not relevant |  | During |
| Tan et al [9] | COVID-19 | IES-R | Mean | HE and LE |  | Not given | 5.85 (9.24) | 9.40 (10.08) |  | During |
| Matsuishi et al 2020 [17] | H1N1 | IES-R | Mean | HE and LE |  | 2.49 (6.63) | 3.1 (8.0) | 2.2 (5.9) |  | During |
| Lee et al 2018 [18] | MERS | IES-R | Mean | HE and LE |  | 26.3 (19.09) | 30.02 (19.55) | 22.33 (17.70) |  | During |
| Bukhari et al 2016 [19] | MERS | IES | Not given | HE and LE |  | Not given | Not given | Not given |  | During |
| Chan &Huak 2004 (doctors) [20] | SARS | IES | Mean | HE and LE |  | Not given | 15.1 (15.8) | 15.4 (15.4) |  | During |
| Chan &Huak 2004 (nurses) [20] | SARS | IES | Mean | HE and LE |  | Not given | 16.8 (12.9) | 16.0 (14.6) |  | During |
| Koh et al 2005 [22] | SARS | IES | Median | HE and LE |  | 16 | Not given | Not given |  | During |
| C.S. Chen et al 2005 [15] | SARS | IES-R | Mean | HE and LE |  | 17.8 (12.4) | 19.1 (14.2) | 13.7 (9.1) |  | During |
| Ho et al 2005 [23] | SARS | CIES-R | Not given | Undifferentiated |  | Not given | Not given | Not given |  | During |
| Chong et al 2004 [21] | SARS | IES | Mean | HE and LE |  | 34.8 (19.7) | 30.6 (15.8) | 35.1 (20.0) |  | During |
| Styra et al 2008 [26] | SARS | IES-R | Not given | HE and LE |  | Not given | Not given | Not given |  | During |
| Su et al 2007 [24] | SARS | DTS-C | Mean | HE and LE |  | Not given | 17.6 (11.9) | 15.2 (12.5) |  | During |
| Lin et al 2007 [27] | SARS | DTS-C | Mean | HE and LE |  | Not given | 26.03 (3.09) | 16.04 (3.26) |  | During |
| Sim et al | SARS | IES-R | Mean | HE and LE |  | Not given | Not given | Not given |  | 9 weeks after |
| Tham et al 2004 (doctors) [28] | SARS | IES | Median | Undifferentiated |  | 9.5 | Not relevant | Not relevant |  | 6 months after |
| Tham et al 2004 (nurses) [28] | SARS | IES | Median | Undifferentiated |  | 15 | Not relevant | Not relevant |  | 6 months after |
| McAlonan et al 2005 [12] | SARS | IES-R | Mean | HE and LE |  | Not given | 17.2 (14.2) | 9.3 (11.2) |  | During & 1 year |
| Maunder et al 2006 [30] | SARS | IES | Median | HE and LE |  | Not given | 11 | 7 |  | 1-2 years after |
| Lancee et al 2008 [31] | SARS | IES | Mean | HE only |  | Not relevant | 12.8 (10.3) | Not relevant |  | 1-2 years after |
| Wu et al 2008/2009 [32][33] | SARS | IES-R | Not given | HE and LE |  | Not given | Not given | Not given |  | 3 years after |

PTSD-SS = PTSD Self Report Scale; IES = Impact of Events Scale; IES-R = Impact of Events Scale – Revised; SASR = Stanford Acute Stress Reaction; CIES-R = Chinese Impact of Event Scale-Revised; Chinese version of the Davidson Trauma Scale (DTS-C)

**Table S5.** All studies reporting averages of validated PTSD symptom scales for healthcare responders for high risk epidemic and pandemic disease outbreaks

**Table S6. Depression averages**

|  |  |  |  |  |  | *Average (SD)* | | |  |  |
| --- | --- | --- | --- | --- | --- | --- | --- | --- | --- | --- |
| *Study* | *Condition* | *Measure* | *Summary stat* | *Sample* |  | *Overall* | *High exposure* | *Low exposure* |  | *Timing* |
| Zhu et al 2020 [7] | COVID-19 | PHQ-9 | Not given | HE and LE |  | Not given | Not given | Not given |  | During |
| Lai et al 2020 [6] | COVID-19 | PHQ-9 | Median | HE and LE |  | 5.0 | 6.0 | 4.0 |  | During |
| Chung & Yeung 2020 [34] | COVID-19 | PHQ-9 | Mean | Undifferentiated |  | 7.6 (No SD) | Not given | Not given |  | During |
| Liang et al [5] | COVID-19 | ZSRAD | Mean | HE and LE |  | Not given | 30.05 (8.16) | 34.09 (8.97) |  | During |
| Z. Liu et al 2020 [3] | COVID-19 | ZSRAD | Not given | HE and LE |  | Not given | Not given | Not given |  | During |
| Sun et al [8] | COVID-19 | SCL-90-A | Mean | HE and LE |  | 1.53 (0.79) | Not given | Not given |  | During |
| Zhang et al 2020 [10] | COVID-19 | PHQ-2 | Mean | HE and LE |  | 1.25 (1.32) | 1.35 (1.37) | 1.18 (1.28) |  | During |
| Tan et al [9] | COVID-19 | DASS-21 | Mean | HE and LE |  | Not given | 2.54 (5.23) | 3.24 (5.07) |  | During |
| Ji et al 2017 [11] (Chinese staff) | Ebola | SCL-90-R-A | Mean | HE only |  | Not relevant | 1.22 (0.31) | Not relevant |  | During |
| Ji et al 2017 [11] (Sierra Leone staff) | Ebola | SCL-90-R-A | Mean | HE only |  | Not relevant | 2.10 (0.78) | 1.96 (0.90) |  | During |
| Chan & Huak 2004 (doctors) [20] | SARS | GHQ-28 D | Mean | HE and LE |  | Not given | 0.6 (1.1) | 0.4 (0.9) |  | During |
| Chan & Huak 2004 (nurses) [20] | SARS | GHQ-28 D | Mean | HE and LE |  | Not given | 0.3 (0.6) | 0.4 (1.0) |  | During |
| McAlonan et al 2005 [12] | SARS | DASS-21 | Mean | HE and LE |  | Not given | 4.9 (4.8) | 2.2 (2.6) |  | During & 1 year |
| C.S. Chen et al 2005[15] | SARS | SCL-90-R-A | Mean | HE and LE |  | 0.65 (0.62) | 0.64 (0.7) | 0.55 (0.47) |  | During |
| R. Chen et al 2006 [13] | SARS | ZSRAD | Mean | HE Only |  | 61 | Not given | Not given |  | During, T1 |
| Sim et al 2004 [25] | SARS | GHQ-28 D | Mean | HE and LE |  | Not given | 0.1 (0.5) | 0.2 (0.9) |  | During |
| Su et al 2007 [24] | SARS | BDI | Mean | HE and LE |  | Not given | 10.2 (6.6) | 6.7 (6.6) |  | During |

PHQ-9 = Patient Health Questionnaire 9; ZSRAD = Zung Self-rating Depression Scale; SCL-90-ASymptom Checklist 90 Depression subscale; BDI = Beck Depression Inventory

**Table S6.** All studies reporting averages of validated anxiety depression scales for healthcare responders for high risk epidemic and pandemic disease outbreaks

**References**

1. Xiao H, Zhang Y, Kong D, Li S, Yang N. The Effects of Social Support on Sleep Quality of Medical Staff Treating Patients with Coronavirus Disease 2019 (COVID-19) in January and February 2020 in China. Med Sci Monit. 2020;26:e923549-1-e923549-8.

2. Liu C, Yang Y, Zhang XM, Xu X, Dou Q-L, Zhang W-W. The prevalence and influencing factors for anxiety in medical workers fighting COVID-19 in China: A cross-sectional survey. preprint. Psychiatry and Clinical Psychology; 2020. doi:10.1101/2020.03.05.20032003.

3. Liu Z, Han B, Jiang R, Huang Y, Ma C, Wen J, et al. Mental Health Status of Doctors and Nurses During COVID-19 Epidemic in China. SSRN Scholarly Paper. Rochester, NY: Social Science Research Network; 2020. https://papers.ssrn.com/abstract=3551329. Accessed 28 Mar 2020.

4. Huang JZ, Han MF, Luo TD, Ren AK, Zhou XP. Mental health survey of 230 medical staff in a tertiary infectious disease hospital for COVID-19. [Chinese]. Zhonghua lao dong wei sheng zhi ye bing za zhi = Zhonghua laodong weisheng zhiyebing zazhi = Chinese journal of industrial hygiene and occupational diseases. 2020;38:E001.

5. Liang Y, Chen M, Zheng X, Liu J. Screening for Chinese medical staff mental health by SDS and SAS during the outbreak of COVID-19. Journal of Psychosomatic Research. 2020;133:110102.

6. Lai J, Ma S, Wang Y, Cai Z, Hu J, Wei N, et al. Factors Associated With Mental Health Outcomes Among Health Care Workers Exposed to Coronavirus Disease 2019. JAMA Netw Open. 2020;3:e203976–e203976.

7. Zhu Z, Xu S, Wang H, Liu Z, Wu J, Li G, et al. COVID-19 in Wuhan: Immediate Psychological Impact on 5062 Health Workers. medRxiv. 2020;:2020.02.20.20025338.

8. Sun N, Xing J, Xu J, Geng LS, Li QY. Study of the mental health status of medical personnel dealing with new coronavirus pneumonia. medRxiv. 2020;:2020.03.04.20030973.

9. Tan BYQ, Chew NWS, Lee GKH, Jing M, Goh Y, Yeo LLL, et al. Psychological Impact of the COVID-19 Pandemic on Health Care Workers in Singapore. Ann Intern Med. 2020. doi:10.7326/M20-1083.

10. Zhang W, Wang K, Yin L, Zhao W, Xue Q, Peng M, et al. Mental Health and Psychosocial Problems of Medical Health Workers during the COVID-19 Epidemic in China. PPS. 2020;:1–9.

11. Ji D, Ji Y-J, Duan X-Z, Li W-G, Sun Z-Q, Song X-A, et al. Prevalence of psychological symptoms among Ebola survivors and healthcare workers during the 2014-2015 Ebola outbreak in Sierra Leone: a cross-sectional study. Oncotarget. 2017;8:12784–91.

12. McAlonan GM, Lee AM, Cheung V, Wong JWS, Chua SE. “Psychological distress and negative appraisals in survivors of severe acute respiratory syndrome (SARS)” and “Severe acute respiratory syndrome (SARS) in Hong Kong in 2003: Stress and psychological impact among frontline healthcare workers”: Comment. Psychological Medicine. 2005;35:459–60.

13. Chen R, Chou K-R, Huang Y-J, Wang T-S, Liu S-Y, Ho L-Y. Effects of a SARS prevention programme in Taiwan on nursing staff’s anxiety, depression and sleep quality: A longitudinal survey. Journal of Nursing Studies. 2006;43:215–25.

14. Iancu I, Strous R, Poreh A, Kotler M, Chelben Y. Psychiatric Inpatients’ Reactions to the SARS Epidemic: An Israeli Survey. Journal of Psychiatry and Related Sciences. 2005;42:258–62.

15. Chen C-S, Wu H-Y, Yang P, Yen C-F. Psychological Distress of Nurses in Taiwan Who Worked During the Outbreak of SARS. PS. 2005;56:76–9.

16. Poon E, Liu KS, Cheong DL, Lee CK, Yam LYC, Tang WN. Impact of severe respiratory syndrome on anxiety levels of front-line health care workers. Hong Kong Med J. 2004;10:325–30.

17. Matsuishi K, Kawazoe A, Imai H, Ito A, Mouri K, Kitamura N, et al. Psychological impact of the pandemic (H1N1) 2009 on general hospital workers in Kobe. Psychiatry and Clinical Neurosciences. 2012;66:353–60.

18. Lee SM, Kang WS, Cho A-R, Kim T, Park JK. Psychological impact of the 2015 MERS outbreak on hospital workers and quarantined hemodialysis patients. Comprehensive Psychiatry. 2018;:123–7.

19. Bukhari EE, Temsah MH, Aleyadhy AA, Alrabiaa AA, Alhboob AA, Jamal AA, et al. Middle East respiratory syndrome coronavirus (MERS-CoV) outbreak perceptions of risk and stress evaluation in nurses. The Journal of Infection in Developing Countries. 2016;10:845–50.

20. Chan AOM, Huak CY. Psychological impact of the 2003 severe acute respiratory syndrome outbreak on health care workers in a medium size regional general hospital in Singapore. Occup Med (Lond). 2004;54:190–6.

21. Chong M-Y, Wang W-C, Hsieh W-C, Lee C-Y, Chiu N-M, Yeh W-C, et al. Psychological impact of severe acute respiratory syndrome on health workers in a tertiary hospital. The British Journal of Psychiatry. 2004;185:127–33.

22. Koh D, Lim MK, Chia SE, Ko SM, Qian F, Ng V, et al. Risk Perception and Impact of Severe Acute Respiratory Syndrome (SARS) on Work and Personal Lives of Healthcare Workers in Singapore What Can We Learn? Medical Care. 2005;43:676–82.

23. Ho SMY, Kwong-Lo RSY, Mak CWY, Wong JS. Fear of Severe Acute Respiratory Syndrome (SARS) Among Health Care Workers. Journal of Consulting and Clinical Psychology. 2005;73:344–9.

24. Su T-P, Lien T-C, Yang C-Y, Su YL, Wang J-H, Tsai S-L, et al. Prevalence of psychiatric morbidity and psychological adaptation of the nurses in a structured SARS caring unit during outbreak: A prospective and periodic assessment study in Taiwan. Journal of Psychiatric Research. 2007;41:119–30.

25. Sim K, Chong PN, Chan YH, Soon WSW. Severe Acute Respiratory Syndrome-Related Psychiatric and Posttraumatic Morbidities and Coping Responses in Medical Staff Within a Primary Health Care Setting in Singapore. Journal of Clinical Psychiatry. 2004;65:1120–7.

26. Styra R, Hawryluck L, Robinson S, Kasapinovic S, Fones C, Gold WL. Impact on health care workers employed in high-risk areas during the Toronto SARS outbreak. Journal of Psychosomatic Research. 2008;64:177–83.

27. Lin C-Y, Peng Y-C, Wu Y-H, Chang J, Chan C-H, Yang D-Y. The psychological effect of severe acute respiratory syndrome on emergency department staff. Emergency Medicine Journal. 2007;24:12–7.

28. Tham K-Y, Tan Y, Loh O, Tan W, Ong M, Tang H. Psychiatric morbidity among emergency department doctors and nurses after the SARS outbreak. Annals of the Academy of Medicine, Singapore. 2004;33:S78-9.

29. Phua DH, Tang HK, Tham KY. Coping Responses of Emergency Physicians and Nurses to the 2003 Severe Acute Respiratory Syndrome Outbreak. Academic Emergency Medicine. 2005;12:322–8.

30. Maunder RG, Lancee WJ, Balderson KE, Bennett JP, Borgundvaag B, Evans S, et al. Long-term Psychological and Occupational Effects of Providing Hospital Healthcare during SARS Outbreak. Emerg Infect Dis. 2006;12:1924–32.

31. Lancee WJ, Maunder RG, Goldbloom DS. Prevalence of Psychiatric Disorders Among Toronto Hospital Workers One to Two Years After the SARS Outbreak. PS. 2008;59:91–5.

32. Wu P, Liu X, Fang Y, Fan B, Fuller CJ, Guan Z, et al. Alcohol Abuse/Dependence Symptoms Among Hospital Employees Exposed to a SARS Outbreak. Alcohol Alcohol. 2008;43:706–12.

33. Wu P, Fang Y, Guan Z, Fan B, Kong J, Yao Z, et al. The psychological impact of the SARS epidemic on hospital employees in China: Exposure, risk perception, and altruistic acceptance of risk. Journal of Psychiatry. 2009;54:302–11.

34. Chung J, Yeung W. Staff Mental Health Self-Assessment During the COVID-19 Outbreak. East Asian Arch Psychiatry. 2020;30:34.

35. Li L, Wan C, Ding R, Liu Y, Chen J, Wu Z, et al. Mental distress among Liberian medical staff working at the China Ebola Treatment Unit: a cross sectional study. Health and Quality of Life Outcomes. 2015;13:156.
