# Supplementary material for: Mental health of clinical staff working in high-risk epidemic and pandemic health emergencies a rapid review of the evidence and living meta-analysis
Source: Soc Psychiatry Psychiatr Epidemiol. 2020 Nov 27;56(1):1–11. doi: 10.1007/s00127-020-01990-x (PMC7691696; doi:10.1007/s00127-020-01990-x)
Supplement: Supplementary file 2 — Supplementary file2 (DOCX 119 KB) [file 127_2020_1990_MOESM2_ESM.docx]

Quantitative studies

| *Study* | *Disease* | *Country* | *Design* | *Participants* | *Measures* | *Key Findings* |
| --- | --- | --- | --- | --- | --- | --- |
| Chung and Yeung, 2020 [1] | COVID-19 | Hong Kong | Cross-sectional, during outbreak | Healthcare staff (N=69) | Patient Health Questionnaire-9 (PHQ-9) | The mean PHQ-9 score was 7.6, with 24 (34.8%) respondents having a score of ≥10 (the cut-off for mild depression) and 10 (14.5%) respondents having a score of ≥15 (the cut-off for moderate depression). |
| Dai et al, 2020 [2] | COVID-19 | China | Cross-sectional, during outbreak | Healthcare workers (N=4357) | Bespoke questions about COVID-19  General Health Questionnaire (GHQ-12) using 0-0-1-1 scoring method | 32.2% of sample involved in frontline treatment of COVID-19 patients. The main concerns of workers were: infection of colleagues (72.5%), infection of family members (63.9%), protective measures (52.3%) and medical violence (48.5%). Only 34.7% of HCWs expressed very worried about the risk of self-infection. Of the 4,357 HCWs, 1,704 (39.1%) had psychological distress (GHQ score above 3).Being female, being in frontline treatment, being in isolation, not volunteering, and having colleagues or family infected predicted higher psychological distress. Job role did not predict distress. |
| Huang, J. et al., 2020 [3] | COVID-19 | China | Cross-sectional,  during outbreak | Medical staff (N=230) in a tertiary infectious disease hospital | Self-rating Anxiety Scale  Post-Traumatic Stress Disorder Self-rating Scale | Rates of severe anxiety, moderate anxiety and mild anxiety were 2.17% (5/230), 4.78% (11/230) and 16.09% (37/230), respectively.The incidence of stress disorder in medical staff was 27.39% (63/230), and the score of PTSD-SS was (42.92 ± 17.88). Nurses and female staff tended to show higher rates.  Cut-offs for caseness unclear |
| Huang, L. et al, 2020 [4] | COVID-19 | China | Cross-sectional, during outbreak | Nurses and nursing college students (N=802) | Bespoke questions about fear, sadness and anger in response to outbreak  Brief COPE | Anxiety, sadness and anger was higher in nurses than nursing college students. Females were higher in anxiety and fear than males. Fear predicted problem-focused coping, anger emotion-focused coping. Closeness to COVID-19 predicted increased anxiety and anger. |
| Lai et al, 2020 [5] | COVID-19 | China | Cross-sectional, during outbreak | Health care workers (N=1257) in hospitals managing COVID-19 patients | Patient Health Questionnaire (PHQ-9)  Generalized Anxiety Disorder scale (GAD-7)  7-item Insomnia Severity Index (ISI)  Impact of Events Scale–Revised (IES-R) | “Nurses, women, frontline health care workers, and those working in Wuhan, China, reported more severe degrees of all measurements of mental health symptoms than other health care workers (eg, median [IQR] Patient Health Questionnaire scores among physicians vs nurses: 4.0 [1.0-7.0] vs 5.0 [2.0-8.0]; P = .007; median [interquartile range {IQR}] Generalized Anxiety Disorder scale scores among men vs women: 2.0 [0-6.0] vs 4.0 [1.0-7.0]; P < .001; median [IQR] Insomnia Severity Index scores among frontline vs second-line workers: 6.0 [2.0-11.0] vs 4.0 [1.0-8.0]; P < .001; median [IQR] Impact of Event Scale–Revised scores among those in Wuhan vs those in Hubei outside Wuhan and those outside Hubei: 21.0 [8.5-34.5] vs 18.0 [6.0-28.0] in Hubei outside Wuhan and 15.0 [4.0-26.0] outside Hubei; P < .001).”  “Frontline health care workers engaged in direct diagnosis, treatment, and care of patients with COVID-19 were associated with a higher risk of symptoms of depression (OR, 1.52; 95% CI, 1.11-2.09; P = .01), anxiety (OR, 1.57; 95% CI, 1.22-2.02; P < .001), insomnia (OR, 2.97; 95% CI, 1.92-4.60; P < .001), and distress (OR, 1.60; 95% CI, 1.25-2.04; P < .001).”  Note: prevalence for staff with symptoms quoted in abstract is for any symptoms (mild range and above). Numbers of above cut-off (typically moderate range >26 and above) are quoted in Table 2. |
| Li, Z. et al, 2020 [6] | COVID-19 | China | Cross-sectional, during outbreak | Front-line nurses (N=234), non-front line nurses (N=292), members of the general public (N=214) | Vicarious Trauma Scale completed on an app | Front-line nurses who came in close contact with patients with COVID-19 reported less vicarious trauma than both non-frontline nurses and the general public – who were not significantly different from each other. |
| Liang et al, 2020 [7] | COVID-19 | China | Cross-sectional, during outbreak | Doctors and nurses from COVID-19-associated departments (N=38)  Doctors and nurses (N=21) from non-COVID-19 departments (Pulmonary and Critical Care, Cardiology, General ICU) | Zung Self-rating Depression Scale (SDS)  Zung Self-rating Anxiety Scale (SAS) | No significant difference between clinical staff in COVID-19 and non-COVID-19 departments.  No difference between older and younger staff. |
| Liu, C-H. et al, 2020 [8] | COVID-19 | China | Cross-sectional, during outbreak | Healthcare workers (N=512) | Bespoke questions about COVID-19  Zung Self-rating Anxiety Scale | 32.03% of workers had had direct contactby treating infected patients. Prevalence of anxiety was 12.5%, with 53 workers suffering from mild (10.35%), seven workers from moderate (1.36%) and four workers from severe anxiety (0.78%). Medical staff who had had direct contact treating infected patients saw higher anxiety scores than those who had not had direct contact. |
| Liu, Z. et al, 2020 [9] | COVID-19 | China | Cross-sectional, during outbreak | Hospital doctors and nurses (N=4679) | WHO 20-item Self-Reporting Questionnaire  Zung Self-rating Anxiety Scale  Zung Self-rating Depression Scale | 44.2% sample worked at departments with high-risk exposure to COVID-19  “The prevalence of psychological distress, anxious symptoms, and depressive symptoms were 15.9% (95% CI 14.8-16.9), 16.0% (95% CI 15.0-17.1), and 34.6% (95% CI 33.2-35.9). “  “Medical staff in middle age group, with divorced or widowed marital status, seldom or not living with family members, working at higher risk department, having experience of treating COVID-19 or infectious diseases had higher risk of psychological distress, while those from infectious hospitals had lower risk of psychological distress. Medical staff from higher-risk departments, general hospitals, or higher-level hospitals had higher risk of anxious symptoms, while younger age group staff, or doctor had lower risk. For depressive symptom, middle age, seldom or not living with family members, or working at a designated hospital for COVID-19 treatment were risk factors, while being a doctor was a protective factor”  “about one-third medical staff have received help from mental health professionals. However, medical staff with mental health problems received less help compared with those without any problems.” |
| Qi et al., 2020 [10] | COVID-19 | China | Cross-sectional, during outbreak | Healthcare workers (N=1306) split into frontline workers (N=801) and non-front-line workers (N=505) | Pittsburgh Sleep Quality Index  Athens Insomnia Scale  Bespoke visual analogue scale of factors influencing sleep quality, work pressure, attention, anxiety, energy, confidence, irritability, stress, impatience, fear, and depression | Front-line medical workers had significantly poorer sleep, and worse anxiety and depression. Severity of COViD-19, work stress, insufficient sleep time and frequent work shifts were linked to poor sleep in all workers. The VAS scales of anxiety and depression were not standardised although mean scores were below the mid-point for all workers. |
| Sun et al, 2020 [11]  (NB: First name on MedRXiv page is Sun, on pdf it’s Xing) | COVID-19 | China | Cross-sectional, during outbreak | Hospital doctors and nurses (N=548) | Bespoke questions about COVID-19  Symptom Checklist-90 (SCL-90) | Factors affecting the mental health and status of medical personnel were degree of suspicion that they were infected when novel coronavirus-related symptoms occurred, level of concern about whether they and their family members were infected, age, whether they had worked in affected areas or hospitals, and whether family members support them working on the front line. Occupation, level of expert is, family and demographic factors did not predict mental health status. Comparison to national norms showed no difference in depression and relatively small differences in other measures. |
| Xiao et al, 2020 [12] | COVID-19 | China | Cross-sectional, during outbreak | Medical staff (N=180) who treated COVID-19 patients | Social Support Rating Scale  Self-Rating Anxiety Scale  General Self-Efficacy Scale  Stanford Acute Stress Reaction scale  Pittsburgh Sleep Quality Index | “Levels of social support for medical staff were significantly associated with self-efficacy and sleep quality and negatively associated with the degree of anxiety and stress. Levels of anxiety were significantly associated with the levels of stress, which negatively impacted self-efficacy and sleep quality. Anxiety, stress, and self-efficacy were mediating variables associated with social support and sleep quality.” |
| Zhu et al, 2020 [13] | COVID-19 | China | Cross-sectional, during outbreak | Healthcare workers (N=5062) | Bespoke questions about COVID-19  Patient Health Questionnaire-9 (PHQ-9)  General Anxiety Disorder-7 (GAD-7)  Impact of Events Scale Revised | “1509 (29.8%), 681 (13.5%) and 1218 (24.1%) HWs reported stress, depression and anxiety symptoms. Women (hazard ratio[HR], 1.31; P=0.032), years of working> 10 years (HR, 2.02; P<0.001), concomitant chronic diseases (HR, 1.51; P<0.001), history of mental disorders (HR, 3.27; P<0.001), and family members or relatives confirmed or suspected (HR, 1.23; P=0.030) were risk factors for stress, whereas care provided by hospital and department administrators(odds ratio [OR], 0.76; P=0.024) and full coverage of all departments with protective measures (OR, 0.69; P=0.004) were protective factors.”  Nurses reported higher levels of stress, anxiety and depression than doctors. Frontline working was associated with stress but not anxiety or depression.  Used >33 cut-off for positive screen on IES-R |
| Tan et al, 2020 [14] | COVID-19 | Singapore | Cross-sectional, during outbreak | Medical and non-medical healthcare staff (N=470) | Depression, Anxiety and Stress Scale (DASS-21)  Impact of Events  Scale-Revised (IES-R) | "Of 500 invited health care workers, 470 (94%) participated in the study; baseline characteristics are shown in Table 1. Sixty-eight (14.5%) participants screened positive for anxiety, 42 (8.9%) for depression, 31 (6.6%) for stress, and 36 (7.7%) for clinical concern of PTSD. The prevalence of anxiety was higher among nonmedical health care workers than medical personnel (20.7% versus 10.8%; adjusted prevalence ratio, 1.85 [95% CI, 1.15 to 2.99]; P = 0.011), after adjustment for age, sex, ethnicity, marital status, survey completion date, and presence of comorbid conditions. Similarly, higher mean DASS-21 anxiety and stress subscale scores and higher IES-R total and subscale scores were observed in nonmedical health care workers" |
| Xu et al, 2020 [15] | COVID-19 | China | Cross-sectional, during outbreak | Surgical medical staff | Undescribed anxiety, depression and dream anxiety scales  “SF-36” (presumably the 36-Item Short Form Survey) | “According to statistics, the anxiety score of surgical staff during the outbreak period was 7.817 ± 2.508, of which 28 were positive (≥8 points). The anxiety score of surgical staff during the non-outbreak period was 5.283 ± 1.738, of which 6 were anxiety positive. The anxiety score of the surgical staff during the outbreak period was significantly higher than that of the surgical staff during the non-outbreak period (t = 6.432, P <0.001). The depression score of surgical staff during the outbreak period was 7.333 ± 2.508, of which 24 were positive for depression (≥8 points). The depression score of surgical staff during the non-outbreak period was 4.933 ± 2.154, of which 7 were positive for depression. Obviously, the depression score of surgical staff during the outbreak was higher than that of surgical staff during the non-outbreak (t = 4.531, P <0.001). At the same time, the dream anxiety score and SF-36 of the surgical staff during the outbreak were significantly higher than those of the surgical staff during the non-outbreak (t = 17.365, P <0.001; t = 1.974, P <0.001).” |
| Zhang et al, 2020 [16] | COVID-19 | China | Cross-sectional, during outbreak | Medical (N=927) and non-medical (N=1255) healthcare workers | Insomnia Severity Index (ISI)  Patient  Health Questionnaire-4 (PHQ-4) including the Generalized  Anxiety Disorder 2-item (GAD-2) and the Patient Health Questionnaire 2-item (PHQ-2)  Symptom Check List-90-  revised (SCL-90-R) | "Compared with nonmedical health workers (n = 1,255), medical health workers (n = 927) had a higher prevalence of insomnia (38.4 vs. 30.5%, p < 0.01), anxiety (13.0 vs. 8.5%, p < 0.01), depression (12.2 vs. 9.5%; p < 0.04), somatization (1.6 vs. 0.4%; p < 0.01), and obsessive-compulsive symptoms (5.3 vs. 2.2%; p < 0.01). They also had higher total scores of ISI, GAD-2, PHQ-2, and SCL-90-R obsessive-compulsive symptoms (p ≤ 0.01). Among medical health workers, having organic disease was an independent factor for insomnia, anxiety, depression, somatization, and obsessive-compulsive symptoms (p < 0.05 or 0.01). Living in rural areas, being female, and being at risk of contact with COVID-19 patients were the most common risk factors for insomnia, anxiety, obsessive-compulsive symptoms, and depression (p < 0.01 or 0.05)." |
| Ji et al, 2017 [17] | Ebola | Sierra Leone | Cross-sectional, during outbreak  Chinese medical staff had before and after measures | Sierra Leone medical staff (N=59), SL logistic staff (N=21), SL medical students (N=22), Chinese medical staff (N=41), Ebola survivors (N=18) | Symptoms Checklist 90-items, Revised (SCL-90-R) | Sierra Leone staff scored considerably higher than Chinese staff on measures of poor mental health. On arrival, Chinese medical staff typically scored Chinese population means for measure of poor mental health except for anxiety. On departure, Chinese medical staff scored below the population mean for the measure of poor mental health. |
| Lehmann et al, 2016 [18] | Ebola | Germany | Cross-sectional, during outbreak | Healthcare workers from 1. isolation unit treating Ebola patient; 2. Ebola research laboratory; 3. Control sample of staff from standard internal medicine wards | Short Form Health Survey (SFHS-12)  Bespoke questionnaire | Comparisons between groups (n1 = 42, n2 = 32, n3 = 12) yielded no significant differences in HrQoL, subjective risk of infection, and most other psychosocial variables. However, the Ebola patient treatment group experienced significantly higher levels of social isolation than both other groups.The best predictors of poor physical and mental HrQoL were perceived lack of knowledge about the Ebola virus disease and fatigue |
| Li, L. et al, 2015 [19] | Ebola | Liberia | Cross-sectional, during outbreak | Nurses and hygienists (N-52) working at the China Ebola Treatment Unit | Symptoms Checklist 90-items, Revised (SCL-90-R) | “Among the work duty subgroups, local medical staff responsible for cleaning and disinfection showed significantly higher levels of obsessive-compulsive (1.68 ± 1.03 versus 1.24 ± 0.78, p < 0.05), anxiety (1.65 ± 0.99 versus 1.16 ± 0.52, p < 0.05), phobic anxiety (1.94 ± 1.19 versus 1.47 ± 1.05, p < 0.05) and PST categories (38.64 ± 16.61 versus 18.73 ± 17.89, p < 0.05) than treatment ward staff, and of anxiety level (1.68 ± 1.03 versus 1.19 ± 0.61, p < 0.05) and PST (38.64 ± 16.61 versus 22.05 ± 15.83, p < 0.05) than observation ward staff” |
| von Strauss et al, 2017 [20] | Ebola | Various in West Africa | Cross-sectional, retrospective | Nurses (N=44) involved in care for Ebola patients | Bespoke questionnaire | “The respondents identified the following needs for improvement: increased mental health and psychosocial support and hands-on coping strategies with focus on pre- and post-deployment; more pre-deployment task-oriented clinical training; and workload reduction, as exhaustion is a risk for safety.” |
| Waterman et al, 2018 [21] | Ebola | Sierra Leone | Single arm intervention study of three-phase peer-delivered CBT-based anxiety and depression treatment | Sierra Leonean staff (N=3273) at Ebola treatment centre | Custom wellbeing scale  Post-Traumatic Stress Checklist  Perceived Stress Scale  Insomnia Severity Index  Generalised Anxiety Disorder 7  Patient Health Questionnaire 9 (PHQ9)  Relationship Questionnaire  Custom behaviour questionnaire | No means or prevalences reported  Improvements in the bespoke wellbeing measure, PTSD, stress, sleep disruption, anxiety, depression and anger by phase 3.  Note, Sierra Leone had been declared Ebola free by the end of phase 2. |
| Austria-Corrales et al, 2011 [22] | H1N1 | Mexico | Cross-sectional, during outbreak | Medical residents (N=99) | MaslachBurnout Inventory | 36.4% (36/99) were above cut-off for burnout on the Maslach scale with those in respiratory medicine reporting highest levels of burnout. |
| Goulia et al, 2010 [23] | H1N1 | Greece | Cross-sectional, during outbreak | Healthcare workers (N=469) of tertiary teaching hospital dealing with H1N1 cases | Bespoke questions on concerns and worries about the ongoing H1N1 pandemic  Cassileth’s Information Styles Questionnaire  General  Health Questionnaire-28 | Level of worry about H1N1 was high and typically focused on risk of infecting family and friends on impact on functional ability. Nursing and auxillary staff reported higher levels of psychological distress than medical staff. The majority of staff reported no GHQ-28 psychological distress, 20.7% reported mild-moderate distress, 6.8% severe distress. |
| Matsuishi et al, 2012 [24]  Same sample used in Imai et al, 2010 [25] | H1N1 | Japan | Cross-sectional, conducted after H1N1 pandemic | Staff (N=1995) at three tertiary teaching hospitals involved in pandemic response | Bespoke questions Impact of Events Scale Revised | Workers in high-risk work environments had higher anxiety, exhaustion and IES score than workers in low-risk work environments. Nurses had higher anxiety, exhaustion and IES score than doctors.  NB: Calculates ‘total’ IES score as item average. Gives no prevalence of above cut-off scoring |
| Alsubaie et al, 2019 [26] | MERS | Saudi Arabia | Cross-sectional, after outbreak | Healthcare workers (N=526) in tertiary teaching hospital | Bespoke SARS questions | “The mean anxiety score was similar for physicians and other HCWs (3/5). However, non physicians expressed higher levels of anxiety toward the risk of transmitting MERS CoV to their families, with an anxiety score of 4/5 compared to 3/5 for the physicians group” |
| Bukhari et al, 2016 [27] | MERS | Saudi Arabia | Cross-sectional, unclear timing, probably during | Healthcare workers (N=386) | Bespoke SARS questions  Impact of events scale (IES) | NB: IES results not reported  “The majority of the participants were females (332; 86.0%), and there were 54 (14.0%) males. Of the 386 respondents, nurses constituted the majority of the respondents (293; 75.9%), and there were 34 doctors (8.8%). The percentage of exposure was found to be greater in those who were working in the intensive care unit (ICU) (89; 23%). There was a significant difference in the worry and fear scale of contracting the MERS-CoV infection between participants who worked in isolation areas, ICUs, and emergency rooms (mean: 3.01 ± 1.1) compared to participants who worked in areas that are less likely to admit and have MERS-CoV suspected or positive cases (mean: 2.77 ± 1.1; p = 0.031. Females were significantly more worried and fearful of contracting the virus compared to males (mean: 2.92 ± 1.1 versus 2.61 ± 1.0, respectively; p = 0.045).” |
| Khalid et al, 2016 [28] | MERS | Saudi Arabia | Cross-sectional, during outbreak | Staff (N=117) at hospital managing MERS outbreak | Bespoke MERS stress questionnaire | Staff reported moderate levels of fear and nervousness but reported their job was a professional and ethical duty. Seeing colleagues in hospital for MERS, fear of making mistakes and fear of infecting family were reported as the most stressful. Positive attitude from colleagues, lack of colleague infection and colleagues’ recovery from MERS were reported as the most stress reducing experiences. Strict use of protective measures was reported the most effect personal coping strategy. |
| Lee, S.M. et al, 2018 [29] | MERS | South Korea | Cross-sectional with high risk group follow-up | Hospital practitioners (N=359) | Impact of Events Scale-Revised | Of all respondents, 230(64.1%) received a score of 18 or higher, indicating the presence ofPTSD-like symptoms, while 183 respondents (51.5%) exceeded thecut-off score of 25 for a diagnosis of PTSD. Healthcare workers who performed MERS-related tasks scored significantly higher on the total IES-R and its subscales  Used IES cut-off of >25 |
| Oh et al, 2017 [30] | MERS | South Korea | Cross-sectional, retrospective | Nurses (N=313) at general hospitals during the MERS outbreak | Modified trauma appraisal questionnaire  Modified questionnaire for professionalism in Korean nurses  Modified questionnaire for professionalism in Korean nurses  Modified Instrument for Predictive Nursing Intention for SARS Patient Care | No significant difference in stress scores between nurses with first-hand or second-hand experience of MERS patients. Intention to provide nursing care to patients with MERS related to lower stress. |
| Park et al, 2018 [31] | MERS | South Korea | Cross-sectional, during outbreak | Nurses (N=187) | Mental component summary of the Short Form-36 (SF-36)  Perceived Stress Scale-10 (PSS-10)  Dispositional Resilience Scale-15 (DRS-15) | “The influences of stigma and hardiness on mental health were partially mediated through stress in nurses working at a hospital during a MERS-CoV epidemic.” |
| Jung et al, 2020 [32] | MERS | South Korea | Cross-sectional, shortly after outbreak | Nurses (N=147) | Impact of Event Scale–Revised Korean version (IES-R-K)  Job Content Questionnaire (K-JCQ)  General Health Questionnaire (GHQ) | "Of the 147 participants, 33.3% were involved in the direct care of the infected patients, whereas 66.7% were involved in the direct care of the suspected patients. More than half (57.1%) of the nurses experienced PTSD, with 25.1% experienced full PTSD and 32.0% with moderate or some level of PTSD. The mean score of turnover intention was 16.3, with the score range of 4 to 20. The multiple regression analysis revealed that PTSD was positively associated with turnover intention, and supervisor support had a strong buffering effect." |
| Bai et al, 2004 [33] | SARS | Taiwan | Cross-sectional, during outbreak | Healthcare workers (N=228) | Bespoke SARS questions | "Seventeen staff members (5 percent) suffered from an acute stress disorder; stepwise multiple logistic regression analysis determined that quarantine was the most related factor. Sixty-six staff members (20 percent) felt stigmatized and rejected in their neighborhood because of their hospital work, and 20 of 218 health care workers (9 percent) reported reluctance to work or had considered resignation." |
| Chan and Huak, 2004 [34] | SARS | Singapore | Cross-sectional, during outbreak | Doctors and nurses (N=661) in hospital managing SARS patients | General Health Questionnaire (GHQ-28)  Impact of Events Scale  Bespoke questionnaire on changes in life priorities andcoping | “In total 177 out of 661 (27%) participants [40 out of 113 (35%) doctors and 137 out of 544 (25%) nurses] had a GHQ 28 score ≥5. Doctors [P = 0.026, odds ratio (OR) = 1.6 and 95% confidence interval (CI) = 1.1–2.5] and single health care workers were at higher risk (P = 0.048, OR = 1.4 and 95% CI = 1.02–2.0) compared to nurses and those who were married. Approximately 20% of the participants had IES scores ≥30, indicating the presence of post-traumatic stress disorder (PTSD)” |
| Chan et al, 2005 [35] | SARS | Hong Kong | Cross-sectional, during outbreak | Nurses (N=1470) | Bespoke SARS questions | “Most nurses (68.3-80.1%) always/often perceived stress from the SARS epidemic. The proportion was the highest in the moderate-risk group (P < 0.001). Most nurses perceived their stress came from work (85.9-95.6%), with 43.8-58.5% perceiving stress from home, and 35-46% perceiving stress from the community. About half (50.7%) of the nurses in the moderate-risk group perceived that they could sometimes/never cope with stress, compared with 45.6% in the high-risk group and 38.9% in the low-risk group (P < 0.001)” |
| Chang et al, 2006 [36] | SARS | Taiwan | Cross-sectional, during outbreak | Medical professionals (N=244) of centres involving in treating SARS patients | Custom questionnaire asking about emotional exhaustion, job tension, social interaction and trust. | Trust in colleagues was associated with lower emotional exhaustion and job tension. |
| Chen, C-S et al, 2005a [37]  NB: Same sample as scale validation study in Chen et al, 2005b | SARS | Taiwan | Cross-sectional, during outbreak | Nurses (N=131) who worked during SARS outbreak.  Compared nurses in units with high risk of exposure (N=65), involuntarily conscripted into high risk units (N=21), those in low risk units(N=45) | Impact of Events Scale (IES)  Symptom Checklist Revised (SCL-90-R) | The highest rate of scoring above IES cut-off was observed in thegroup that worked in a high-risk unit, and the conscriptedgroup experienced the most severe SCL-90-R measured distress on average.  14 nurses (11%) screened positive on the IES. Positive cases were highest in the high-risk group (11 nurses, or 17%), followed bythe conscripted group (two nurses, or 10 %) and the control group(one nurse, or 2%).  Used >35 cut-off for IES-R |
| Chen, R. et al, 2006 [38] | SARS | Taiwan | Longitudinal. Four time points. 1. Before SARS patients; 2. Two weeks after programme start; 3. One month after programme start; 4. One month after decommissioning SARS response | Nursing staff (N=116) in designated SARS response hospital after implementation of prevention plan | Zung anxiety scale  Zung depression scale  Pittsburgh sleep quality index | All reported very high levels of pre-SARS job stress. Anxiety and depression reduced from moderate to minimal over time. Sleep quality remained poor throughout.  Staff who did not volunteer for SARS patient care reported worse anxiety and depression. |
| Chong et al, 2004 [39] | SARS | Taiwan | Cross-sectional, during outbreak  Split into two time points depending on when the scales were completed | Healthcare workers (N=1310) | Bespoke questions on exposure to SARS  Impact of Events Scale (IES)  Chinese Health Questionnaire (CHQ) | “The study period was arbitrarily divided into two phases for analysis: the initial shock and reaction phase (12 May to 6 June), when the situation was chaotic and the number of patients infected with SARS was escalating; and the repair or reorientation phase (7 June to 27 June), when no new infections occurred and the situation was brought under control.” |
| Chua et al, 2004 [40] | SARS | Hong Kong | Cross-sectional, during outbreak | Healthcare workers (N=271)  Comparison group of non-healthcare workers (N=342) | Bespoke questions about SARS  Perceived Stress Scale (PSS-10 | “Strikingly, HCWs were not more stressed than healthy control subjects (HCWs mean 18.6, control subjects mean 18.3; Mann–Whitney U,P> 0.9). We observed that PSS score and negative psychological effects from SARS were highly significantly correlated (Spearman’s rho = 0.4,P< 0.001).”  “HCWs who were confident about infection control (74%,n= 179) had lower stress levels(independent samples ttest, 2-tailed,P= 0.001) and fewer negative effects (independent samples ttest, 2-tailed,P=0.004).” |
| Fiksenbaum et al, 2006 [41] | SARS | Canada | Cross-section, during outbreak | Nurses (N=333) across all specialities | Maslach Burnout Inventory  State-Trait Anger Expression Inventory  Survey of Perceived Organisational Support  Modified vigor scale  Bespoke question about avoidance, trust in equipment/infection control, contact with SARS patients, experience of quarantine | No scale means / prevalences reported  “Results of path analysis revealed that working conditions contributed significantly to an increase in perceived SARS threat, which led to increased emotional exhaustion and state anger. Positive feedback was directly and positively related to organizational support. Higher levels of organizational support predicted lower perceived SARS threat, emotional exhaustion, and state anger” |
| Grace et al, 2005 [42] | SARS | Canada | Cross-sectional, during outbreak | Physicians (N=193) at hospitals in which SARS patients were treated | Bespoke questionnaire asking about health, attitudes and perceptions toward SARS; coping, symptoms, effects on personal relationships, changes to work resultingfrom SARS outbreak | Physicians providing direct care to SARSpatients more commonly reported psychological distress (45.7%, N=16) than among not providingdirect care (17.7%, N=28). Thirty-five respondents (18.1%) reported experiencingnew distressing psychological symptoms that they attributedto working during the SARS outbreaks. Physicians reported both positive and negative aspects of working in the outbreak. |
| Ho et al, 2005 [43] | SARS | Hong Kong | Cross-sectional, during outbreak | Staff at hospitals managing SARS patients (N=82) and hospital staff who recovered from SARS (N=97) | Bespoke questions asking about SARS fear, SARS self-efficacy  For SARS recovered workers, the Chinese Impact of Event Scale—  Revised only related to SARS events | “participants in both samples had equal, if not more, concern about infecting others (especially family members) than being self-infected”. “Participants with lower self-efficacy tended to have higher fear related toSARS. Fear related to SARS was also correlated positively with posttraumatic stress symptoms among respondents of Sample 2 (recovered staff).” |
| Iancu et al, 2005 [44] | SARS | Israel | Cross-sectional, during outbreak | Inpatients (N=30) and staff (N=30) at a psychiatric hospital | Modified Spielberger Anxiety Scale  Bespoke questionnaire items measuring fear of SARS | Staff had lower anxiety than psychiatric patients but the groups did not differ in SARS related anxiety. |
| Koh et al, 2005 [45] | SARS | Singapore | Cross-sectional, during outbreak | Healthcare workers (N=10,511) in hospitals with and without SARS patients during outbreak | Bespoke questions asking about perception of exposure to SARS, perceived risk of infection, and impact of the SARS outbreak on personal and work life  Impact of Events Scale | Two thirds (66%) reported feeling “at great risk of exposure to SARS”. Predictors of increased stress at work included SARS-affected hospitals, daily exposure toSARS patients, occupation (nurses), married with children, and high IES score.  “Clinical staff (doctors and nurses), staff in daily contact with SARS patients, and staff from SARS-affected institutions expressed significantly higher levels of anxiety”  A total of 56% reported feeling “more stressed at work,” whereas 53% experienced “increase in workload.”  On the positive side, the majority of respondents felt appreciated by their hospital/clinic/employer (82%) and by society (77%).69.5% of respondents accepted the risk of contracting SARS as part and parcel of their job  IES means / prevalence not reported |
| Lancee et al, 2008 [46] | SARS | Canada | Follow-up, incidence study | Healthcare workers (completed CAPS N=139; completed SCID N=133) who took part in Maunder et al (2006) study | Clinician-Administered PTSD Scale (CAPS). Structured Clinical Interview for DSM-IV (SCID) | "This study found that one to two years after the resolution of the SARS outbreak in Canada, the incidence of new episodes of major depression among health care workers who were still working was 4% (five of 133 participants) and the incidence of new-onset PTSD was 2%. The incidence of any new onset of a psychiatric disorder was 5%. These incidence rates appear to be lower than those found in the general population. For example, the estimated annual incidence of major depression in Canada for women aged 25 to 44 has been reported to be 4.5%, and for women aged 45 to 64 it is 4.1%. The incidence of depression also appears to be lower than the recently reported one-year rate of 9% for Canadian nurses.” |
| Lee, S-H. et al, 2005 [47] | SARS | Taiwan | Cross-sectional, during outbreak | Female nurses (N=26) in SARS nursing team | Bespoke ‘SARS Team Questionnaire’ | Worries largely centred on being a risk to others, rather than experiencing risk themselves. Good team cohesion, psychiatric support and effective equipment and working environment were considered effective in reducing stress. Nurses reported using both practical (infection control related) and psychological coping. |
| Lin et al, 2007 [48] | SARS | Taiwan | Cross-sectional, during outbreak | Emergency department doctors and nurses (N=66)  Control group of doctors and nurses from medium risk psychiatric ward (N=26) | Davidson Trauma Scale-Chinese version (DTS-C)  Chinese Health Questionnaire-12 (CHQ-12) | “86 of 92 (93.5%) medical staff considered the SARS outbreak to be a traumatic experience. The DTSC scores of staff in the emergency department and in the psychiatric ward were significantly different (p = 0.04). No significant difference in CHQ score was observed between the two groups. Emergency department staff had more severe PTSD symptoms than staff in the psychiatric ward.” |
| Liu, X. et al, 2012 [49]  Same sample as Wu et al, 2008 | SARS | China | Cross-sectional, retrospective 3 years after outbreak | Hospital staff (N=549) of hospital affected by SARS outbreak | As above | “The results of multinomial regression analyses showed that, with other relevant factors controlled for, being single, having been quarantined during the outbreak, having been exposed to other traumatic events before SARS, and perceived SARS-related risk level during the outbreak were found to increase the odds of having a high level of depressive symptoms 3 years later. Altruistic acceptance of risk during the outbreak was found to decrease the odds of high post-outbreak depressive symptom levels.” |
| Lung et al, 2009 [50] | SARS | Taiwan | Cross-sectional, during outbreak with one year follow-up | Healthcare workers (N=127) | Chinese Health Questionnaire (CHQ-12)  Eysenck Personality Questionnaire  Parental Bonding Instrument | “Healthcare workers that had mental symptoms at follow-up reported the symptoms were associated with daily-life stress and not the SARS crisis. The physicians had more somatic symptoms than nurses, suggesting different professions have different impact on mental health. Additionally, individual’s early maternal attachment and neuroticism were found to have greater effect on mental health of life-threatening stress” |
| Marjanovic et al, 2007 [51]  (same data set as above) | SARS | Canada | Cross-sectional, during outbreak | Nurses (N=333) across all specialities | Maslach Burnout Inventory  State-Trait Anger Expression Inventory  Survey of Perceived Organisational Support  Modified vigor scale  Bespoke question about avoidance, trust in equipment/infection control, contact with SARS patients, experience of quarantine | No scale means / prevalences reported  Organizational support and trust in equipment/infection control predicted lower rates of emotional exhaustion.  State anger was positively correlated to avoidance behavior, contact with SARS patients, and greater time in quarantine; and negatively related to vigor, organizational support, and trust in equipment/infection control initiatives. |
| Maunder et al, 2006 [52] | SARS | Canada | Follow-up,  13 to 26 months after the SARS outbreak | Healthcare workers (N=769) from Toronto hospitals involved and Hamilton hospitals not involved in managing SARS patients | Impact of Events Scale (IES)  Kessler Psychological Distress Scale (K10)  Maslach Burnout Inventory (MBI-EE)  A subset of participants answered bespoke questions on SARS-related perception of stigma and interpersonal avoidance; adequacy of training, protection, and support; and job stress | “During the study period (13–25 months after the SARS outbreak), Toronto HCWs reported significantly higher levels of burnout (Toronto median score 19, interquartile range 10–29; Hamilton 16, 9– 23, p = 0.019), psychological distress (Toronto 15, 12–19; Hamilton 13, 11–17, p<0.001), and posttraumatic stress (Toronto 11, 4–21; Hamilton 7, 0–19, p<0.001). To make these differences more clinically meaningful, the prevalence of high scores was calculated (Table 4). The prevalence of the following functional indicators of distress since the SARS outbreak was higher in Toronto HCWs: decrease in patient contact and work hours, increase in substance use and other traits that interfere with function, and more days off work (Table 4). Of the 7 adverse outcomes reported in Table 4, Toronto HCWs were more likely to be experiencing >1 problem (Toronto 68.1% vs. Hamilton 50.1%, p<0.001)) and were almost twice as likely to be experiencing multiple (>2) problems (Toronto 44.0% vs. Hamilton 22.5%, p<0.001).”  Used >26 cut-off for IES |
| McAlonan et al, 2007 [53] | SARS | Hong Kong | Cross-sectional study of healthcare workers at two time points (not follow-up) | Healthcare workers in 2003 (N = 176) and 2004 (N=184) | Perceived Stress Scale (PSS-10)  Depression, Anxiety and Stress Scale (DASS-21)  Impact of Events  Scale-Revised (IES-R) | "In 2003, high-risk health care workers had elevated stress levels (PSS-10 score = 17.0) that were not significantly different from levels in low-risk health care worker control subjects (PSS-10 score = 15.9). More high-risk health care workers reported fatigue, poor sleep, worry about health, and fear of social contact, despite their confidence in infection-control measures. By 2004, however, stress levels in the high-risk group were not only higher (PSS-10 score = 18.6) but also significantly higher than scores among low-risk health care worker control subjects (PSS-10 score = 14.8, P < 0.05). In 2004, the perceived stress levels in the high-risk group were associated with higher depression, anxiety, and posttraumatic stress scores (P < 0.001). Posttraumatic stress scores were a partial mediator of the relation between the high risk of exposure to SARS and higher perceived stress." |
| Nickell et al, 2004 [54] | SARS | Canada | Cross-sectional, during outbreak | Healthcare and non-healthcare workers (N=2001) at hospital managing SARS patients | Bespoke questions on occupation and work history; closed and open-ended questions about the respondent’s concerns about SARS; closed and open-ended questions about the use and effects of SARS precautionary measures  General Health Questionnaire (GHQ-12) | Nurses most likely to score about GHQ cut-off (45.1%), allied health care professionals (33.3%), doctors (17.4%) and staff not working in patient care (18.9%).  Reported negative effects of the SARS outbreak included financial losses, being treated differently by people because of working in a hospital and changes to personal and familial lifestyle.  Over half of the respondents (1161 [58.0%]) also reported at least 1 positive effect (Table 4). A total of 493 (41.1%) felt there was an increased awarenessof disease control, 317 (26.4%) found the SARS outbreak to be a learning experience, and 285 (23.8%) felt an increased sense of togetherness and cooperation. Other positive aspects included being less busy than usual and feeling a greater appreciation of life and work.  4 factors as being significantly associated with the presence of emotional distress, as identified with the GHQ-12: being a nurse (adjusted OR 2.8, 95% CI 1.5–5.5), part-time employment status (adjusted OR 2.6, 95% CI 1.2–5.4), lifestyle affected by the SARS outbreak (adjusted OR 2.2, 95% CI 1.4–3.5) and ability to do one’s job affected by the precautionary measures (adjusted OR 2.9, 95% CI 1.9–4.6). |
| Phua et al, 2008 [55]  NB: Same data as Tham et al. | SARS | Singapore | Cross-sectional, 6 months after outbreak | Emergency department doctors and nurses (N=96) | Coping Orientation  to Problems Experienced (COPE)  Impact of Event Scale (IES)  General Health Questionnaire 28 (GHQ-28) | “The respondents reported a preference for problem-focused and emotion-focused coping measures. The physicians chose humor as a coping response significantly more frequently (p , 0.001) than nurses, scoring 9.61/16 (95% CI = 8.52 to 10.69), compared with the nurses’ score of 7.05/16 (95% CI = 6.28 to 7.83). The Filipino HCWs turned to religion as a coping response significantly more frequently (p , 0.001) than the non-Filipino HCWs, scoring 14.38/16 (95% CI = 13.33 to 15.42), compared with 9.93/16 (95% CI = 9.00 to 10.87) for the non-Filipinos. Psychiatric morbidity was 17.7% on the IES and 18.8% on the GHQ 28, with the trend for physicians to report lower psychiatric morbidity.” |
| Poon et al, 2004 [56] | SARS | Hong Kong | Cross-sectional, during outbreak | Healthcare workers (N=1926) | State-Trait Anxiety Inventory (STAI)  Modified Maslach Burnout Inventory | “Anxiety scores ranged from a minimum of 20 to a maximum of 80 and mean scores were highest among workmen (55.9 [SD, 9.7]), followed by health care assistants (52.9[8.6]), nurses (52.0 [9.8]), doctors (47.8 [11.1]), allied health workers (47.8 [10.9]), technicians (47.8 [9.8]), administrative staff (47.1 [10.6]), and transport workers (46.4 [9.4]). Scores among workmen, health care assistants, and nurses were significantly higher than scores among doctors(P<0.001 for each pairwise ttest) and administrative staff controls (P<0.001).” |
| Sim et al, 2004 [57] | SARS | Singapore | Cross-sectional, during outbreak | Doctors and nurses (N=277) | General Health Questionnaire 28 (GHQ-28)  Impact of Event Scale-Revised (IES-R)  Brief Coping Orientation  to Problems Experienced (COPE) | See Table 2 for high- versus low-exposure results.  “HCWs who were confident about infection control (74%,n= 179) had lower stress levels (independent samplest test, 2-tailed,P= 0.001) and fewer negative effects (independent samples ttest, 2-tailed,P=0.004).”  NB: idiosyncratic IES-R scoring: “Two scores were calculated from the IES-R, namely a continuous score (total and subscales) and a dichotomous categorization of high versus low level of posttraumatic symptoms. When calculating the dichotomous scores, we considered symptoms present if the respondentsreported that they had been at least moderately distressed by the symptoms in the previous week (score of at least 2 on a scale of 0–4)” |
| Styra et al, 2008 [58] | SARS | Canada | Cross-sectional, during outbreak with control groups (oncology, general medicine, cardiology unit, general surgery, multiorgan transplant surgery within the same hospital system) sampled from non-SARS services | Healthcare workers (N=248) | Bespoke SARS questions  Impact of Event  Scale-Revised (IES-R) | “Seventeen staff members (5 percent) suffered from an acute stress disorder; stepwise multiple logistic regression analysis determined that quarantine was the most related factor. Sixty-six staff members (20 percent) felt stigmatized and rejected in their neighborhood because of their hospital work, and 20 of 218 health care workers (9 percent) reported reluctance to work or had considered resignation.” |
| Su et al, 2007 [59] | SARS | Taiwan | Longitudinal, during outbreak | Nurse participants were from two SARS units (regular SARS N=44] and SARS ICU N=26) and two non-SARS  units (Neurology N=15] and CCU N=17). | Bespoke SARS attitude scale  Beck depression inventory (BDI)  Pittsburgh sleep quality index (PSQI)  Sheehan’s disability scale  Family APGAR index (family functioning measure) | "Results showed that depression (38.5% vs. 3.1%) and insomnia (37% vs. 9.7%) were, respectively, greater in the SARS unit nurses than the non-SARS unit nurses. No difference between these two groups was found in the prevalence of post-traumatic stress symptoms (33% vs. 18.7%), yet, three unit subjects (SARS ICU, SARS regular and Neurology) had significantly higher rate than those in CCU (29.7% vs. 11.8%, respectively) (p < 0.05). For the SARS unit nurses, significant reduction in mood ratings, insomnia rate and perceived negative feelings as well as increasing knowledge and understanding of SARS at the end of the study (all p < 0.001) indicated that a gradual psychological adaptation had occurred. The adjustment of nurses in the more structured SARS ICU environment, where nurses care for even more severely ill patients, may have been as good or better than that of nurses in the regular SARS unit." |
| Tam et al, 2004 [60] | SARS | Hong Kong | Cross-sectional, during outbreak | Healthcare workers (N=652) | Bespoke SARS and coping questions  Chinese version of the  12-item General Health Questionnaire (GHQ) using 0-0-1-1 scoring method | “Four hundred and forty four participants (68%) reported ‘significant’ or ‘severe’ levels of job-related stress during the outbreak and 205 (32%) reported stress levels as ‘mild’ or ‘moderate’.”  “A total of 56.7% of the participants scored above the threshold on GHQ (3 or more). Cases were more likely to have higher levels of job-related stress and poor self-rated physical health, and be less willing to work in SARS units. Female workers and nursing professionals were more likely to be cases. Perceived inadequacy of all support items except ‘appreciation from the community’ were significantly associated with psychological morbidity” |
| Tham et al, 2004 [61] | SARS | Singapore | Cross-sectional, 6 months after outbreak | Emergency department doctors and nurses (N=96) | Impact of Event Scale (IES)  General Health Questionnaire (GHQ 28) | “Fewer doctors reported post-event and psychiatric morbidity compared to nurses, with 5 (13.2%) doctors and 12 (20.7%) nurses scoring ≥26 on IES, 6 (15.8%) doctors and 12 (20.7%) nurses scoring ≥5 on GHQ 28. The doctors reported a median of 9.5 (range 0-47) on IES and 0 (range 0-11) on GHQ 28. The nurses reported a median of 15 (range 0-61) on IES and 1 (range 0-25) on GHQ 28.” |
| Wong, TW. et al, 2005 [62] | SARS | Hong Kong | Cross-sectional, shortly after outbreak | Healthcare workers (N=466) | Bespoke 18-item question to measure SARS related distress  Chinese version of the Brief Cope questionnaire | "A total of 1260 questionnaires were sent out and the response rate was approximately 37%. The mean overall distress level was 6.19 out of a 10-point scale. The mean overall distress levels for doctors, nurses and HCA were 5.91, 6.52 and 5.44, respectively (F(2,420) = 6.47, P < 0.005). The overall distress level for nurses was significantly higher than for HCA (P <0.005) but not doctors. The overall distress level was highly and significantly correlated with the six sources of distress: vulnerability/loss of control (r2 = 0.68); health of self (r2 = 0.62); spread of virus (r2 = 0.60); health of family and others (r2 = 0.59); changes in work (r2 = 0.46); being isolated (r2 = 0.45)." |
| Wong, W.C.W. et al, 2004 [63] | SARS | Hong Kong | Cross-sectional, retrospective | GPs (N=183) working during the SARS outbreak | Bespoke questions asking about medical practice, protective measures and SARS-related anxieties. | "On a scale of 10, the anxiety scores were less than 5 (midpoint) in all parameters with worry about the family (3.44) achieved the lowest score. Female doctors were more worried about infecting their families (2.16 versus 3.67; p,0.05) and perceived high anxiety as a source of infection by their families (3.16 versus 4.69; p,0.05) whereas young and middle aged doctors found their quality of life more affected than their older colleagues (3.67 and 3.35 versus 5.55; p,0.05). However, exposures to SARS and working districts had no impact on their anxiety levels." |
| Wu et al, 2008 [64] | SARS | China | Cross-sectional, 3 years after outbreak | Healthcare workers (N = 549) | Bespoke SARS questions  Seven questions regarding alcohol abuse/dependence symptoms, adapted from the National Household Survey on Drug Abuse (NHSDA)  Impact of Events Scale Revised (IES-R)  Center for Epidemiologic Studies Depression Scale (CES-D)  Bespoke questions about exposure to other traumatic events and coping | "Current alcohol abuse/dependence symptom counts 3 years after the outbreak were positively associated with having been quarantined, or worked in high-risk locations such as SARS wards, during the outbreak. However, having had family members or friends contract, SARS was not related to alcohol abuse/dependence symptom count. Symptoms of PTS and of depression, and having used drinking as a coping method, were also significantly associated with increased alcohol abuse/dependence symptoms. The relationship between outbreak exposure and alcohol abuse/dependence symptom count remained significant even when sociodemographic and other factors were controlled for. When the intrusion, avoidance and hyperarousal PTS symptom clusters were entered into the model, hyperarousal was found to be significantly associated with alcohol abuse/dependence symptoms" |
| Wu et al, 2009 [65]  Same sample as Wu et al, 2008 | SARS | China | Cross-sectional, retrospective 3 years after outbreak | Hospital staff (N=549) of hospital affected by SARS outbreak | Custom questions on SARS exposure, non-SARS trauma exposure, During-outbreak perceptions of SARS-related risks.  Impact of Events Scale Revised | About 10% of employees were above cut-off for post-traumatic stress symptoms since outbreak. SARS exposure at work, quarantining, and a relative orfriend getting SARS were all strongly associated with high symptom levels. Retrospectively remembered perceived levels of SARS risk were positively related with their current levelsof fear. |

Qualitative studies

| *Study* | *Disease* | *Country* | *Design* | *Participants* | *Measures* | *Findings* |
| --- | --- | --- | --- | --- | --- | --- |
| Cunningham et al, 2017 [66] | Ebola | Various in West Africa | Qualitative, retrospective | Expatriate clinical staff who cared for Ebola patients (N=19) | Interview | Themes: memorialising, advocacy, self-reflection, and camaraderie |
| Hewlett and Hewlett, 2005 [67] | Ebola | Uganda, Republic of Congo | Qualitative, retrospective | Nurses and healthcare workers who worked in the Ebola outbreak (example sample size unclear) | Interview | “Three key themes emerged from the interviews: (a)lack of protective gear, basic equipment, and other resourcesnecessary to provide care, especially during the early phasesof the outbreaks; (b) stigmatization by family, coworkers, andcommunity; and (c) exceptional commitment to the nursingprofession in a context where the lives of the health careworkers were in jeopardy” |
| Meyer et al, 2018 [68] | Ebola | United States | Qualitative, retrospective | Healthcare workings (N=77) involved in Ebola response | Interview | Themes: Stress of caring for Ebola patients, isolation and stigma from other hospital staff, friends and family. Support from hospital leadership and mental health programmes help mitigate stress and improve morale, rigorous training and involvement in infection control measures helped alleviate stress. |
| Raven et al, 2018 [69] | Ebola | Sierra Leone | Qualitative | Healthcare workers caring for Ebola patients (N=25), other staff involved in Ebola response (N=19) | Interview | Relevant Themes:  Impact of the outbreak on health workers (breakdown of trust was reported between neighbours /communities and health workers; isolation from families; fear of being infected; trauma from watching colleagues die; economic hardship; increased stress and workload)  Coping strategies (Sense of duty to serve their country and their communities; Peer and family support; Social media platform ; Religion) |
| McMahon et al, 2016 [70] | Ebola | Sierra Leone | Qualitative, during outbreak | Healthcare workers (N=35) | Interview | “A theme articulated across interviews was Ebola’s destruction of social connectedness and sense of trust within and across health facilities, communities and families. Providers described feeling lonely,ostracized, unloved, afraid, saddened and no longer respected. They also discussed restrictions onbehaviors that enhance coping including attending burials and engaging in physical touch (hugging,handshaking, sitting near, or eating with colleagues, patients and family members). Providers described infection prevention measures as necessary but divisive because screening booths and protective equipment inhibited bonding or ‘suffering with’ patients.” |
| Smith et al, 2017 [71] | Ebola | United States | Qualitative, retrospective | Staff (N=21) who participated in the care of the Ebola patients in | Interview | “(1) positive experiences were emotional while challenges were technical; (2) a significant percentage of workers encountered interpersonal stressors, with 29% of respondents having feelings of isolation, 33% having alterations in home life, and 25% experiencing at least 1 episode of discrimination; (3) physicians and nurses had stressors primarily related to patient care; (4) mental health was an important supportive service, with 45% of respondents using behavioral health counseling; and (5) working in the biocontainment unit during activation was more stressful than everyday work for 60% of respondents.” |
| Wong E.L.Y. et al, 2012 [72] | H1N1 | Hong Kong | Qualitative,  during outbreak | Healthcare workers (N=10) working in H1N1 isolation wards | Interview | Themes: Willingness to stay in post and work as needed (professionalism, duty); Concerns (support from management seen as positive; risk of infection and vaccination; frequent policy changes and unclear criteria for case management; high patient turnover rate; poor facility layout; role stress). |
| Chiang et al, 2007 [73] | SARS | Taiwan | Qualitative, retrospective | Nurses (N=21) who cared for patients during the SARS outbreak | Interview | Themes of self-preservation and fear, viewing themselves in their work with patients and colleagues, and self-improvement. |
| Chung et al, 2005 [74] | SARS | Hong Kong | Qualitative, retrospective | Nurses (N=8) who cared for patients during the SARS outbreak | Interview | Themes: A myriad of emotions in caring for SARS patients, uncertainty, revisiting the ‘taken for granted’ features  of nursing. “Initially, emotional turmoil wasexperienced by the participants, who unanimously describedthis as the low point in their new experience” |
| Shih et al, 2009 [75] | SARS | Taiwan | Qualitative, retrospective | Nurse leaders (N=70) from hospitals involved in managing SARS patients | Interview | “Five stages arose in the participants’ involvement against severe acute respiratory syndrome over 12 weeks: facing shock and chaos; searching for reliable sources to clarify myths; developing and adjusting nursing care; supporting nurses and their clients; and rewarding nurses” |

Anecdotal accounts of group response

| *Study* | *Disease* | *Country* | *Design* | *Participants* | *Measures* | *Findings* |
| --- | --- | --- | --- | --- | --- | --- |
| Chen, Q. et al, 2020 [76] | COVID-19 | China | Anecdotal | Healthcare workers | None | “Nurses showed excitability, irritability, unwillingness to rest, and signs of psychological distress, but refused any psychological help.”  “Many staff mentioned that they did not need a psychologist, but needed more rest without interruption and enough protective supplies.” |
| Chan-Yeung, 2004 [77] | SARS | Hong Kong | Anecdotal,  retrospective | Healthcare workers | None | Hospital workers felt lonely and isolated. Anxiety and resentment regarding high risk procedures. |
| Maunder et al, 2003 [78] | SARS | Canada | Anecdotal, retrospective | SARS clinical staff and mental health providers | Anecdotal | “Prominent among the varied responses of individualstaff members were themes of fear, anxiety, anger and frustration.Many expressed conflict between their roles ashealth care provider and parent, feeling on one hand altruismand professional responsibility and, on the other hand,fear and guilt about potentially exposing their families toinfection.”  “…spikes of anxiety occurredin association with several events: when isolation precaution nrocedures changed, when infectious disease staff enteredquarantine or treatment, when health care workerswere admitted with an unclear source of infection, whenone of the SARS-unit nurses developed a fever (not due toSARS) and when a discharged patient with SARS was readmittedwith fever. Staff reported fatigue, insomnia, irritabilityand decreased appetite.” |
| Kwek et al, 2004 [79]  Occasionally indexed as Khee et al, 2004 | SARS | Singapore | Anecdotal, cross-sectional | Healthcare workers who attended support groups | Anecdotal | “Two main trends were observed in this study. The first observation made was a dynamic development of specific behaviors. That is fear, anger, and blame being the main emotions experienced at the beginning of the outbreak. The groups that were assessed after the death toll had risen experienced a great sense of grief and loss. The second trend observed was the development of serious issues after specific events had occurred, for example, a significant sense of grief and frustration after the death of a colleague.” |

**References**

1. Chung J, Yeung W (2020) Staff Mental Health Self-Assessment During the COVID-19 Outbreak. East Asian Arch Psychiatry 30:34

2. Dai Y, Hu G, Xiong H, et al (2020) Psychological impact of the coronavirus disease 2019 (COVID-19) outbreak on healthcare workers in China. medRxiv 2020.03.03.20030874. https://doi.org/10.1101/2020.03.03.20030874

3. Huang JZ, Han MF, Luo TD, et al (2020) Mental health survey of 230 medical staff in a tertiary infectious disease hospital for COVID-19. [Chinese]. Zhonghua lao dong wei sheng zhi ye bing za zhi = Zhonghua laodong weisheng zhiyebing zazhi = Chinese journal of industrial hygiene and occupational diseases 38:E001. http://dx.doi.org/10.3760/cma.j.cn121094-20200219-00063

4. Huang L, Xu F ming, Liu H rong (2020) Emotional responses and coping strategies of nurses and nursing college students during COVID-19 outbreak. medRxiv 2020.03.05.20031898. https://doi.org/10.1101/2020.03.05.20031898

5. Lai J, Ma S, Wang Y, et al (2020) Factors Associated With Mental Health Outcomes Among Health Care Workers Exposed to Coronavirus Disease 2019. JAMA Netw Open 3:e203976–e203976. https://doi.org/10.1001/jamanetworkopen.2020.3976

6. Li Z, Ge J, Yang M, et al (2020) Vicarious traumatization in the general public, members, and non-members of medical teams aiding in COVID-19 control. Brain Behav Immun. https://doi.org/10.1016/j.bbi.2020.03.007

7. Liang Y, Chen M, Zheng X, Liu J (2020) Screening for Chinese medical staff mental health by SDS and SAS during the outbreak of COVID-19. Journal of Psychosomatic Research 133:110102. https://doi.org/10.1016/j.jpsychores.2020.110102

8. Liu C, Yang Y, Zhang XM, et al (2020) The prevalence and influencing factors for anxiety in medical workers fighting COVID-19 in China: A cross-sectional survey. medRxiv 2020.03.05.20032003. https://doi.org/10.1101/2020.03.05.20032003

9. Liu Z, Han B, Jiang R, et al (2020) Mental Health Status of Doctors and Nurses During COVID-19 Epidemic in China. Social Science Research Network, Rochester, NY

10. Qi J, Xu J, Li B, et al (2020) The Evaluation of Sleep Disturbances for Chinese Frontline Medical Workers under the Outbreak of COVID-19. medRxiv 2020.03.06.20031278. https://doi.org/10.1101/2020.03.06.20031278

11. Sun N, Xing J, Xu J, et al (2020) Study of the mental health status of medical personnel dealing with new coronavirus pneumonia. medRxiv 2020.03.04.20030973. https://doi.org/10.1101/2020.03.04.20030973

12. Xiao H, Zhang Y, Kong D, et al (2020) The Effects of Social Support on Sleep Quality of Medical Staff Treating Patients with Coronavirus Disease 2019 (COVID-19) in January and February 2020 in China. Med Sci Monit 26:e923549-1-e923549-8. https://doi.org/10.12659/MSM.923549

13. Zhu Z, Xu S, Wang H, et al (2020) COVID-19 in Wuhan: Immediate Psychological Impact on 5062 Health Workers. medRxiv 2020.02.20.20025338. https://doi.org/10.1101/2020.02.20.20025338

14. Tan BYQ, Chew NWS, Lee GKH, et al (2020) Psychological Impact of the COVID-19 Pandemic on Health Care Workers in Singapore. Ann Intern Med. https://doi.org/10.7326/M20-1083

15. Xu J, Xu Q, Wang C, Wang J (2020) Psychological status of surgical staff during the COVID-19 outbreak. Psychiatry Res. https://doi.org/10.1016/j.psychres.2020.112955

16. Zhang W, Wang K, Yin L, et al (2020) Mental Health and Psychosocial Problems of Medical Health Workers during the COVID-19 Epidemic in China. PPS 1–9. https://doi.org/10.1159/000507639

17. Ji D, Ji Y-J, Duan X-Z, et al (2017) Prevalence of psychological symptoms among Ebola survivors and healthcare workers during the 2014-2015 Ebola outbreak in Sierra Leone: a cross-sectional study. Oncotarget 8:12784–12791. https://doi.org/10.18632/oncotarget.14498

18. Lehmann M, Bruenahl CA, Addo MM, et al (2016) Acute Ebola virus disease patient treatment and health-related quality of life in health care professionals: A controlled study. Journal of Psychosomatic Research 83:69–74. https://doi.org/10.1016/j.jpsychores.2015.09.002

19. Li L, Wan C, Ding R, et al (2015) Mental distress among Liberian medical staff working at the China Ebola Treatment Unit: a cross sectional study. Health and Quality of Life Outcomes 13:156. https://doi.org/10.1186/s12955-015-0341-2

20. von Strauss E, Paillard-Borg S, Holmgren J, Saaristo P (2017) Global nursing in an Ebola viral haemorrhagic fever outbreak: before, during and after deployment. Glob Health Action 10:1371427. https://doi.org/10.1080/16549716.2017.1371427

21. Waterman S, Hunter ECM, Cole CL, et al (2018) Training peers to treat Ebola centre workers with anxiety and depression in Sierra Leone. Journal of Social Psychiatry 64:156–165. https://doi.org/10.1177/0020764017752021

22. Austria-Corrales F, Cruz-Valdés B, Kiengelher LH-, et al (2011) Burnout syndrome among medical residents during the Influenza A H1N1 sanitary contigency in Mexico. Gac Med Mex 147:97–103

23. Goulia P, Mantas C, Dimitroula D, et al (2010) General hospital staff worries, perceived sufficiency of information and associated psychological distress during the A/H1N1 influenza pandemic. BMC Infectious Diseases 10:322. https://doi.org/10.1186/1471-2334-10-322

24. Matsuishi K, Kawazoe A, Imai H, et al (2012) Psychological impact of the pandemic (H1N1) 2009 on general hospital workers in Kobe. Psychiatry and Clinical Neurosciences 66:353–360. https://doi.org/10.1111/j.1440-1819.2012.02336.x

25. Imai H, Matsuishi K, Ito A, et al (2010) Factors associated with motivation and hesitation to work among health professionals during a public crisis: a cross sectional study of hospital workers in Japan during the pandemic (H1N1) 2009. BMC Public Health 10:672. https://doi.org/10.1186/1471-2458-10-672

26. Alsubaie S, Temsah MH, Al-Eyadhy AA, et al (2019) Middle East Respiratory Syndrome Coronavirus epidemic impact on healthcare workers’ risk perceptions, work and personal lives. The Journal of Infection in Developing Countries 13:920–926. https://doi.org/10.3855/jidc.11753

27. Bukhari EE, Temsah MH, Aleyadhy AA, et al (2016) Middle East respiratory syndrome coronavirus (MERS-CoV) outbreak perceptions of risk and stress evaluation in nurses. The Journal of Infection in Developing Countries 10:845–850. https://doi.org/10.3855/jidc.6925

28. Khalid I, Khalid TJ, Qabajah MR, et al (2016) Healthcare Workers Emotions, Perceived Stressors and Coping Strategies During a MERS-CoV Outbreak. Clin Med Res 14:7–14. https://doi.org/10.3121/cmr.2016.1303

29. Lee SM, Kang WS, Cho A-R, et al (2018) Psychological impact of the 2015 MERS outbreak on hospital workers and quarantined hemodialysis patients. Comprehensive Psychiatry 123–127. https://doi.org/10.1016/j.comppsych.2018.10.003

30. Oh N, Hong N, Ryu DH, et al (2017) Exploring Nursing Intention, Stress, and Professionalism in Response to Infectious Disease Emergencies: The Experience of Local Public Hospital Nurses During the 2015 MERS Outbreak in South Korea. Asian Nursing Research 11:230–236. https://doi.org/10.1016/j.anr.2017.08.005

31. Park J-S, Lee E-H, Park N-R, Choi YH (2018) Mental health of nurses working at a government-designated hospital during a MERS-CoV outbreak: A cross-sectional study. Archives of Psychiatric Nursing 32:2–6. https://doi.org/10.1016/j.apnu.2017.09.006

32. Jung H, Jung SY, Lee MH, Kim MS (2020) Assessing the Presence of Post-Traumatic Stress and Turnover Intention Among Nurses Post–Middle East Respiratory Syndrome Outbreak: The Importance of Supervisor Support: Workplace Health & Safety. https://doi.org/10.1177/2165079919897693

33. Bai Y, Lin C-C, Lin C-Y, et al (2004) Survey of stress reactions among health care workers involved with the SARS outbreak. Psychiatric Services 55:1055–1057. https://doi.org/10.1176/appi.ps.55.9.1055

34. Chan AOM, Huak CY (2004) Psychological impact of the 2003 severe acute respiratory syndrome outbreak on health care workers in a medium size regional general hospital in Singapore. Occup Med (Lond) 54:190–196. https://doi.org/10.1093/occmed/kqh027

35. Chan SSC, Leung GM, Tiwari AFY, et al (2005) The Impact of Work-related Risk on Nurses During the SARS Outbreak in Hong Kong. Family & Community Health 28:274–287

36. Chang K, Gotcher DF, Chan M (2006) Does Social Capital Matter When Medical Professionals Encounter the SARS Crisis in a Hospital Setting. Health Care Management Review 31:26–33

37. Chen C-S, Wu H-Y, Yang P, Yen C-F (2005) Psychological Distress of Nurses in Taiwan Who Worked During the Outbreak of SARS. PS 56:76–79. https://doi.org/10.1176/appi.ps.56.1.76

38. Chen R, Chou K-R, Huang Y-J, et al (2006) Effects of a SARS prevention programme in Taiwan on nursing staff’s anxiety, depression and sleep quality: A longitudinal survey. Journal of Nursing Studies 43:215–225. https://doi.org/10.1016/j.ijnurstu.2005.03.006

39. Chong M-Y, Wang W-C, Hsieh W-C, et al (2004) Psychological impact of severe acute respiratory syndrome on health workers in a tertiary hospital. The British Journal of Psychiatry 185:127–133. https://doi.org/10.1192/bjp.185.2.127

40. Chua SE, Cheung V, Cheung C, et al (2004) Psychological Effects of the SARS Outbreak in Hong Kong on High-Risk Health Care Workers. Can J Psychiatry 49:391–393. https://doi.org/10.1177/070674370404900609

41. Fiksenbaum L, Marjanovic Z, Greenglass ER, Coffey S (2006) Emotional Exhaustion and State Anger in Nurses Who Worked During the Sars Outbreak: The Role of Perceived Threat and Organizational Support. Canadian Journal of Community Mental Health 25:89–103. https://doi.org/10.7870/cjcmh-2006-0015

42. Grace SL, Hershenfield K, Robertson E, Stewart DE (2005) The Occupational and Psychosocial Impact of SARS on Academic Physicians in Three Affected Hospitals. Psychosomatics 46:385–391. https://doi.org/10.1176/appi.psy.46.5.385

43. Ho SM, Kwong-Lo RS, Mak CW, Wong JS (2005) Fear of severe acute respiratory syndrome (SARS) among health care workers. Journal of consulting and clinical psychology 73:344

44. Iancu I, Strous R, Poreh A, et al (2005) Psychiatric Inpatients’ Reactions to the SARS Epidemic: An Israeli Survey. Journal of Psychiatry and Related Sciences 42:258–262

45. Koh D, Lim MK, Chia SE, et al (2005) Risk Perception and Impact of Severe Acute Respiratory Syndrome (SARS) on Work and Personal Lives of Healthcare Workers in Singapore: What Can We Learn? Medical Care 43:676–682. https://doi.org/10.1097/01.mlr.0000167181.36730.cc

46. Lancee WJ, Maunder RG, Goldbloom DS (2008) Prevalence of Psychiatric Disorders Among Toronto Hospital Workers One to Two Years After the SARS Outbreak. PS 59:91–95. https://doi.org/10.1176/ps.2008.59.1.91

47. Lee S-H, Juang Y-Y, Su Y-J, et al (2005) Facing SARS: Psychological impacts on SARS team nurses and psychiatric services in a Taiwan general hospital. General Hospital Psychiatry 27:352–358. https://doi.org/10.1016/j.genhosppsych.2005.04.007

48. Lin C-Y, Peng Y-C, Wu Y-H, et al (2007) The psychological effect of severe acute respiratory syndrome on emergency department staff. Emergency Medicine Journal 24:12–17. https://doi.org/10.1136/emj.2006.035089

49. Liu X, Kakade M, Fuller CJ, et al (2012) Depression after exposure to stressful events: Lessons learned from the severe acute respiratory syndrome epidemic. Comprehensive Psychiatry 53:15–23. https://doi.org/10.1016/j.comppsych.2011.02.003

50. Lung F-W, Lu Y-C, Chang Y-Y, Shu B-C (2009) Mental symptoms in different health professionals during the SARS attack: A follow-up study. Psychiatric Quarterly 80:107–116. https://doi.org/10.1007/s11126-009-9095-5

51. Marjanovic Z, Greenglass ER, Coffey S (2007) The relevance of psychosocial variables and working conditions in predicting nurses’ coping strategies during the SARS crisis: An online questionnaire survey. International Journal of Nursing Studies 44:991–998. https://doi.org/10.1016/j.ijnurstu.2006.02.012

52. Maunder RG, Lancee WJ, Balderson KE, et al (2006) Long-term Psychological and Occupational Effects of Providing Hospital Healthcare during SARS Outbreak. Emerg Infect Dis 12:1924–1932. https://doi.org/10.3201/eid1212.060584

53. McAlonan GM, Lee AM, Cheung V, et al (2007) Immediate and Sustained Psychological Impact of an Emerging Infectious Disease Outbreak on Health Care Workers: The Canadian Journal of Psychiatry. https://doi.org/10.1177/070674370705200406

54. Nickell LA, Crighton EJ, Tracy CS, et al (2004) Psychosocial effects of SARS on hospital staff: survey of a large tertiary care institution. CMAJ 170:793–798. https://doi.org/10.1503/cmaj.1031077

55. Phua DH, Tang HK, Tham KY (2005) Coping Responses of Emergency Physicians and Nurses to the 2003 Severe Acute Respiratory Syndrome Outbreak. Academic Emergency Medicine 12:322–328. https://doi.org/10.1197/j.aem.2004.11.015

56. Poon E, Liu KS, Cheong DL, et al (2004) Impact of severe respiratory syndrome on anxiety levels of front-line health care workers. Hong Kong Med J 10:325–330

57. Sim K, Chong PN, Chan YH, Soon WSW (2004) Severe Acute Respiratory Syndrome-Related Psychiatric and Posttraumatic Morbidities and Coping Responses in Medical Staff Within a Primary Health Care Setting in Singapore. Journal of Clinical Psychiatry 65:1120–1127. https://doi.org/10.4088/JCP.v65n0815

58. Styra R, Hawryluck L, Robinson S, et al (2008) Impact on health care workers employed in high-risk areas during the Toronto SARS outbreak. Journal of Psychosomatic Research 64:177–183. https://doi.org/10.1016/j.jpsychores.2007.07.015

59. Su T-P, Lien T-C, Yang C-Y, et al (2007) Prevalence of psychiatric morbidity and psychological adaptation of the nurses in a structured SARS caring unit during outbreak: A prospective and periodic assessment study in Taiwan. Journal of Psychiatric Research 41:119–130. https://doi.org/10.1016/j.jpsychires.2005.12.006

60. Tam CWC, Pang EPF, Lam LCW, Chiu HFK (2004) Severe acute respiratory syndrome (SARS) in Hong Kong in 2003: stress and psychological impact among frontline healthcare workers. Psychological Medicine 34:1197–1204. https://doi.org/10.1017/S0033291704002247

61. Tham K-Y, Tan Y, Loh O, et al (2004) Psychiatric morbidity among emergency department doctors and nurses after the SARS outbreak. Annals of the Academy of Medicine, Singapore 33:S78-9. https://doi.org/10.1177/102490790501200404

62. Wong TW, Yau JKY, Chan CLW, et al (2005) The psychological impact of severe acute respiratory syndrome outbreak on healthcare workers in emergency departments and how they cope. European Journal of Emergency Medicine 12:13–18

63. Wong WCW, Lee A, Tsang KK, Wong SYS (2004) How did general practitioners protect themselves, their family, and staff during the SARS epidemic in Hong Kong? Journal of Epidemiology & Community Health 58:180–185. https://doi.org/10.1136/jech.2003.015594

64. Wu P, Liu X, Fang Y, et al (2008) Alcohol Abuse/Dependence Symptoms Among Hospital Employees Exposed to a SARS Outbreak. Alcohol Alcohol 43:706–712. https://doi.org/10.1093/alcalc/agn073

65. Wu P, Fang Y, Guan Z, et al (2009) The psychological impact of the SARS epidemic on hospital employees in China: Exposure, risk perception, and altruistic acceptance of risk. Journal of Psychiatry 54:302–311

66. Cunningham T, Rosenthal D, Catallozzi M (2017) Narrative medicine practices as a potential therapeutic tool used by expatriate Ebola caregivers. Intervention 15:106–119

67. Hewlett BL, Hewlett BS (2005) Providing Care and Facing Death: Nursing During Ebola Outbreaks in Central Africa. J Transcult Nurs 16:289–297. https://doi.org/10.1177/1043659605278935

68. Meyer D, Kirk Sell T, Schoch-Spana M, et al (2018) Lessons from the domestic Ebola response: Improving health care system resilience to high consequence infectious diseases. American Journal of Infection Control 46:533–537. https://doi.org/10.1016/j.ajic.2017.11.001

69. Raven J, Wurie H, Witter S (2018) Health workers’ experiences of coping with the Ebola epidemic in Sierra Leone’s health system: a qualitative study. BMC Health Services Research 18:251. https://doi.org/10.1186/s12913-018-3072-3

70. McMahon SA, Ho LS, Brown H, et al (2016) Healthcare providers on the frontlines: a qualitative investigation of the social and emotional impact of delivering health services during Sierra Leone’s Ebola epidemic. Health Policy Plan 31:1232–1239. https://doi.org/10.1093/heapol/czw055

71. Smith MW, Smith PW, Kratochvil CJ, Schwedhelm S (2017) The Psychosocial Challenges of Caring for Patients with Ebola Virus Disease. Health Secur 15:104–109. https://doi.org/10.1089/hs.2016.0068

72. Wong ELY, Wong SYS, Lee N, et al (2012) Healthcare workers’ duty concerns of working in the isolation ward during the novel H1N1 pandemic. Journal of Clinical Nursing 21:1466–1475. https://doi.org/10.1111/j.1365-2702.2011.03783.x

73. Chiang H-H, Chen M-B, Sue I-L (2007) Self-State of Nurses in Caring for Sars Survivors. Nurs Ethics 14:18–26. https://doi.org/10.1177/0969733007071353

74. Chung BPM, Wong TKS, Suen ESB, Chung JWY (2005) SARS: caring for patients in Hong Kong. Journal of Clinical Nursing 14:510–517. https://doi.org/10.1111/j.1365-2702.2004.01072.x

75. Shih F-J, Turale S, Lin Y-S, et al (2009) Surviving a life-threatening crisis: Taiwan’s nurse leaders’ reflections and difficulties fighting the SARS epidemic. Journal of Clinical Nursing 18:3391–3400. https://doi.org/10.1111/j.1365-2702.2008.02521.x

76. Chen Q, Liang M, Li Y, et al (2020) Mental health care for medical staff in China during the COVID-19 outbreak. Lancet Psychiatry 7:e15–e16. https://doi.org/10.1016/s2215-0366(20)30078-x

77. Chan-Yeung M (2004) Severe acute respiratory syndrome (SARS) and healthcare workers. Int J Occup Environ Health 10:421–7. https://doi.org/10.1179/oeh.2004.10.4.421

78. Maunder R, Hunter J, Vincent L, et al (2003) The immediate psychological and occupational impact of the 2003 SARS outbreak in a teaching hospital. Canadian Medical Association Journal 168:1245–1251

79. Khee KS, Lee LB, Chai OT, et al (2004) The psychological impact of SARS on health care providers. Critical Care and Shock 100–106
