# Supplementary material for: Mental health of clinical staff working in high-risk epidemic and pandemic health emergencies a rapid review of the evidence and living meta-analysis
Source: Soc Psychiatry Psychiatr Epidemiol. 2020 Nov 27;56(1):1–11. doi: 10.1007/s00127-020-01990-x (PMC7691696; doi:10.1007/s00127-020-01990-x)
Supplement: Supplementary file 3 — Supplementary file3 (DOCX 23 KB) [file 127_2020_1990_MOESM3_ESM.docx]

**Risk of bias assessments for studies included in prevalence tables or meta-analyses**

**Cross-sectional studies**

Based on criteria at:

Risk of Bias in Cross-Sectional Surveys of Attitudes and Practices

<https://www.evidencepartners.com/wp-content/uploads/2017/09/Risk-of-Bias-Instrument-for-Cross-Sectional-Surveys-of-Attitudes-and-Practices.pdf>

1. Is the source population representative of the population of interest?
2. Is the response rate adequate?
3. Is there little missing data?
4. Is the survey clinically sensible face validity? [Face validity]
5. Is there any evidence for the reliability and validity of the survey instrument?

Risk of bias: High / Low; NR = Not reported

| *Study* | *Representative sample* | *Adequate response rate* | *Missing data* | *Face validity* | *Use of validated scales* |
| --- | --- | --- | --- | --- | --- |
| C. Liu et al 2020 | Low | Low | High (NR) | High (NR) | Low |
| C.S. Chen et al 2005 | High | High (NR) | High (NR) | High (NR) | Low |
| Chan & Huak 2004 | Low | Low | High (NR) | High (NR) | Low |
| Chong et al 2004 | Low | High | Low | High (NR) | Low |
| Chung & Yeung, 2020 | Low | High | High (NR) | High (NR) | Low |
| J. Z. Huang et al 2020 | - | - | - | - | Low |
| Jung et al 2020 | High | High (NR) | High (NR) | High (NR) | Low |
| Lai et al 2020 | Low | Low | High (NR) | High (NR) | Low |
| Lancee et al 2008 | High | High | High (NR) | High (NR) | Low |
| Lin et al 2007 | High | High (NR) | High (NR) | High (NR) | Low |
| Matsuishi et al 2012 | Low | High | Low | High (NR) | Low |
| Maunder et al 2006 | Low | High | High (NR) | High (NR) | Low |
| McAlonan et al 2005 | Low | Low | Low | High (NR) | Low |
| Poon et al 2004 | High | Low | High (NR) | High (NR) | Low |
| S.M. Lee et al 2018 | Low | High | High (NR) | High (NR) | Low |
| Sim et al 2004 | Low | High (NR) | High (NR) | High (NR) | Low |
| Tan et al 2020 | Low | High (NR) | High (NR) | High (NR) | Low |
| Tham et al 2004 | Low | Low | High (NR) | High (NR) | Low |
| Wu et al 2008 | Low | Low | High (NR) | High (NR) | Low |
| Z. Liu et al 2020 | High | Unclear | Low | High (NR) | Low |
| Zhang et al 2020 | Low | High (NR) | High (NR) | High (NR) | Low |
| Zhu et al 2020 | Low | Low | Low | High (NR) | Low |

**Case-control studies**

Criteria at:

Tool to Assess Risk of Bias in Case Control Studies

<https://www.evidencepartners.com/wp-content/uploads/2017/09/Tool-to-Assess-Risk-of-Bias-in-Case-Control-Studies.pdf>

1. Can we be confident in the assessment of exposure?
2. Can we be confident that cases had developed the outcome of interest and controls had not?
3. Were the cases (those who were exposed and developed the outcome of interest) properly selected?
4. Were the controls (those who were exposed and did not develop the outcome of interest) properly selected?
5. Were cases and controls matched according to important prognostic variables or was statistical adjustment carried out for those variables?

| *Study* | *Assessment of exposure* | *Case / control outcome differentiation* | *Case selection* | *Control selection* | *Matching* |
| --- | --- | --- | --- | --- | --- |
| C.S. Chen et al 2005 | Low | Low | High | High | High |
| Lin et al 2007 | Low | Low | High | High | High |
| Maunder et al 2006* | High | Low | High | High | High |

*High risk subset follow-up only so included here as case-control study

**Cohort studies**

Tool to Assess Risk of Bias in Cohort Studies

<https://www.evidencepartners.com/wp-content/uploads/2017/09/Tool-to-Assess-Risk-of-Bias-in-Cohort-Studies.pdf>

1. Was selection of exposed and non-exposed cohorts drawn from the same population?
2. Can we be confident in the assessment of exposure?
3. Can we be confident that the outcome of interest was not present at start of study?
4. Did the study match exposed and unexposed for all variables that are associated with the outcome of interest or did the statistical analysis adjust for these prognostic variables?
5. Can we be confident in the assessment of the presence or absence of prognostic factors?
6. Can we be confident in the assessment of outcome?
7. Was the follow up of cohorts adequate?
8. Were co-interventions similar between groups?

| *Study* | *Selection* | *Exposure assessment* | *Outcome not present at start* | *Matching* | *Assessment prognostic factors* | *Assessment outcome* | *Adequate follow-up* | *Similar co-interventions* |
| --- | --- | --- | --- | --- | --- | --- | --- | --- |
| Ji et al 2017 | High | Low | High | High | Low | High | Low | Low |
| Li et al 2015 | High | Low | High | High | Low | High | Low | Low |
| Su et al 2007 | Low | Low | High | High | Low | High | High | Low |
